# Predicting habitat suitability for Townsend's big‐eared bats across California in relation to climate change

**DOI:** 10.1002/ece3.9641

**Published:** 2022-12-15

**Authors:** Natalie M. Hamilton, Michael L. Morrison, Leila S. Harris, Joseph M. Szewczak, Scott D. Osborn

**Affiliations:** ^1^ Department of Rangeland, Wildlife and Fisheries Management Texas A&M University College Station Texas USA; ^2^ Department of Wildlife, Fish, and Conservation Biology University of California, Davis Davis California USA; ^3^ California Polytechnic University‐Humboldt Arcata California USA; ^4^ California Department of Fish and Wildlife, Wildlife Diversity Program Sacramento California USA

**Keywords:** bats, climate change, range shifts, species distribution model

## Abstract

Effective management decisions depend on knowledge of species distribution and habitat use. Maps generated from species distribution models are important in predicting previously unknown occurrences of protected species. However, if populations are seasonally dynamic or locally adapted, failing to consider population level differences could lead to erroneous determinations of occurrence probability and ineffective management. The study goal was to model the distribution of a species of special concern, Townsend's big‐eared bats (*Corynorhinus townsendii*), in California. We incorporate seasonal and spatial differences to estimate the distribution under current and future climate conditions. We built species distribution models using all records from statewide roost surveys and by subsetting data to seasonal colonies, representing different phenological stages, and to Environmental Protection Agency Level III Ecoregions to understand how environmental needs vary based on these factors. We projected species' distribution for 2061–2080 in response to low and high emissions scenarios and calculated the expected range shifts. The estimated distribution differed between the combined (full dataset) and phenologically explicit models, while ecoregion‐specific models were largely congruent with the combined model. Across the majority of models, precipitation was the most important variable predicting the presence of *C. townsendii* roosts. Under future climate scenarios, distribution of *C. townsendii* is expected to contract throughout the state, however suitable areas will expand within some ecoregions. Comparison of phenologically explicit models with combined models indicates the combined models better predict the extent of the known range of *C. townsendii* in California. However, life‐history‐explicit models aid in understanding of different environmental needs and distribution of their major phenological stages. Differences between ecoregion‐specific and statewide predictions of habitat contractions highlight the need to consider regional variation when forecasting species' responses to climate change. These models can aid in directing seasonally explicit surveys and predicting regions most vulnerable under future climate conditions.

## Graphical abstract

The goal of our study was to model the current distribution of Townsend's big‐eared bats (*Corynorhinus townsendii*) and estimate range shifts under future climate conditions at different temporal and geographic scales. We built species distribution models in present and future conditions using all records from statewide roost surveys and by subsetting this data to seasonal colonies and to Environmental Protection Agency Level III ecoregions. As a whole, the range of *C. townsendii* is expected to decrease throughout California, but within some ecoregions suitable areas will increase, highlighting the need to consider intraspecific variation when building species distribution models.

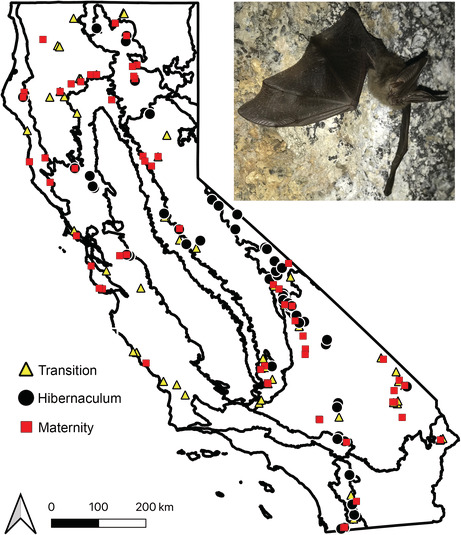

## 1 INTRODUCTION

Over the last century, the earth's climate has changed, including a warming atmosphere and changes in the frequency and intensity of precipitation (Masson‐Delmotte et al., 2021). Major impacts of climate change on biodiversity include increased extinction rate, range shifts, habitat fragmentation, and increased dispersal resistance (reviewed in Habibullah et al., 2021). There is already evidence of species' ranges changing, with research pointing toward major contractions, expansions, and shifts in distributions globally (Amorim et al., 2014; Chen et al., 2011; Freeman et al., 2018). Species distribution models (also known as ecological niche models and habitat suitability models) are popular tools used to predict species' range shifts under various climate and land use change scenarios (Guisan et al., 2013; Razgour et al., 2016). These models use environmental variables and known species occurrences to predict a species' distribution over larger geographic scales. Predicting spatial changes in species' ranges through species distribution modeling can help develop targeted conservation plans by identifying areas that will be able to sustain a species despite climate change, or by aiding in the prioritization of areas for field surveys of rare taxa (Guisan et al., 2013; Zanini et al., 2009).

The variables and processes that are important in explaining the distribution and persistence of species are scale dependent; they can change from one area to the next due to regional differences in biotic and abiotic factors, historical and present land use, and/or local adaptation of populations (Deppe & Rotenberry, 2008 and references within, Bay et al., 2018; Ervin & Holly, 2011; Neubaum & Aagaard, 2022; Razgour et al., 2019; Urbanowicz et al., 2019). However, in species distribution models, the relationship between distribution and predictive environmental variables is assumed to be constant across a species' range (Reed et al., 2011). If populations of a species are locally adapted, excluding geographic population‐level differences could lead to erroneous distribution predictions and inappropriate management decisions as local response to climate change could differ throughout a species' range (Hällfors et al., 2016; Oldfather, 2019). Refining the spatial scale of models, for example, modeling by ecoregion subdivisions, can improve predictions by capturing this intraspecific variation in climate tolerance (Chardon et al., 2020; Ferraz et al., 2012; Hällfors et al., 2016; Smith et al., 2019; Urbanowicz et al., 2019).

Seasonal differences in the distribution of a species are another source of variation to consider when building species distribution models. Species' phenological stages can have vastly different ecological needs and many species of birds and mammals migrate to meet these needs, thus occupying different geographic areas and niches depending on the season (Avgar et al., 2014; Fortuna et al., 2009). Failing to separate species distribution models based on breeding and non‐breeding occurrences assumes that species have similar climatic needs during these periods. Studies on long‐distance migratory animals have shown that different climatic or landscape variables drive species distribution in the suitability breeding and non‐breeding (winter) habitat (i.e., Hayes et al., 2015; Morganti et al., 2017). Additionally, there is a growing body of evidence that supports the same patterns in species that have local seasonal movements (Beumer et al., 2019; Smeraldo et al., 2018). When modeling potential future distribution of species that migrate locally, such as temperate bats, seasonally explicit distribution models may be used to understand if habitat will continue to be suitable for both breeding and non‐breeding needs.

Bats (Chiroptera Blumenbach, 1779) are the second‐most diverse mammalian order with ~1400 species, representing almost a fifth of mammal species (Frick et al., 2019; Mammal Diversity Database, 2022). They provide important ecosystem services such as pollination, seed dispersal, and pest control (Boyles et al., 2011; Jones et al., 2009; Kunz et al., 2011; Maas et al., 2016). Many bat species are at risk of population decline from largely anthropogenic factors including habitat loss, mortality at wind farms, and climate change, therefore many bat species must be actively managed for recovery (Festa et al., 2022; Frick et al., 2019; Voigt & Kingston, 2016). Fine‐scale distribution maps can help prioritize management activities for bats by predicting potential refugia, guiding survey efforts, or providing insights on population connectivity (reviewed in Razgour et al., 2016). Climate change is predicted to cause varying responses in bat species—modeling studies predict both positive (e.g., range expansions and population growth) and negative (e.g., range contractions and population decline) responses, and monitoring studies confirm range shifts have already occurred in some bat species (Ancillotto et al., 2016; Festa et al., 2022; Loeb & Winters, 2013; Piccioli Cappelli et al., 2021; Voigt & Kingston, 2016; Zamora‐Gutierrez et al., 2018).

Our study focused on California populations of Townsend's big‐eared bats, *Corynorhinus townsendii* (Cooper, 1837). This species occurs across the western United States, Canada, and Mexico, with isolated populations in the central and eastern United States. There are currently five recognized subspecies of *Corynorhinus townsendii*; *C. t. australis*, *C. t. ingens*, *C.t. pallescens*, *C.t. townsendii*, and *C. t. virginianus*. The two eastern subspecies (*C. t. ingens* and *C. t. virginianus*) are federally listed endangered species while two western subspecies (*C. t. townsendii* and *C. t. pallescens*) are listed as species of Special Concern or sensitive by state and federal agencies, including the California Department of Fish and Wildlife and also classified as high priority for study by the Western Bat Working Group (California Department of Fish and Wildlife, 2019; Pierson et al., 1999). As *C. townsendii* requires special management attention, building accurate habitat models is essential to promote their conservation. This species occurs throughout the entire state of California, occupying coastal, desert, and mountain ecoregions and roosts in caves, mines, tree hollows, or anthropogenic structures with cavern like features (Fellers & Pierson, 2002; Harris et al., 2019; Mazurek, 2004). During the summer, reproductively active female bats roost in maternity colonies, where they give birth to and raise their one young of the year, and adult males tend to roost singly or in small groups. In the fall, mark–recapture data show maternity colonies disperse as bats travel to hibernacula, which are composed of bats from two to six maternity colonies as well as males (M. L. Morrison, unpublished data). Like many temperate bat species, reproductive female *C. townsendii* select habitat at lower elevations during pregnancy and lactation, for stable temperatures and increased food availability necessary for increased energetic needs, and use latitudinal or elevational migration to find hibernacula with suitable temperatures for extended torpor during the winter (Gruver & Keinath, 2006; reviewed in McGuire & Boyle, 2013). There is also evidence that *C*. *townsendii* exhibit high roost fidelity, where most individuals return to the same summer and winter roosts each year (Anderson et al., 2018; Clark et al., 1996; Sherwin et al., 2000). Because *C. townsendii* have different roosts for phenological stages, we can model the roost‐type separately to understand changes in environmental requirements for different life‐history stages. Additionally, because this species occurs across the diverse ecoregions of California, it is appropriate for understanding how geographic scale of the model affects the predicted distribution, as ecoregions potentially represent areas of local adaptation (due to different environmental characteristics in each region; Pease et al., 2022, Smith et al., 2019). The average temperature in California has increased by approximately 1.11°C since the early 20th century, with warming projected to continue (Frankson et al., 2022). However, warming across the state has not been uniform, suggesting that some ecoregions are experiencing more accelerated effects of climate change—for example, monthly minimums in the Sierra Nevada have increased by about 3°C in the past 100 years (Thorne et al., 2006). Flooding, drought, and wildfires are ecosystem disturbances, influenced by climate conditions, and are also predicted to increase in the next century (Frankson et al., 2022). Due to this predicted temperature change and increase in environmental disturbances in the state, many species, such as *C. townsendii*, could face local extinction, thus identification of potential refugia is critical to conservation efforts in the state.

Our study objectives were to: (1) model the present and future distributions of *C. townsendii* considering two climate change scenarios and determine the limiting climatic or geographic variables in the present, (2) assess the percentage of expansion/contraction in the range of *C. townsendii* and determine where these shifts occur, and (3) assess how these projections vary between different seasonal and spatial scales. Understanding how predicted occurrence (presumably reflective of environmental needs) varies across time (maternity, hibernacula, and transition roosts) and space (different ecoregions) could be critical for helping managers and surveyors pinpoint appropriate areas for conservation actions.

## 2 METHODS

### 2.1 Study area and survey data

The study area covers the U.S. state of California, which has steep environmental gradients that support an array of species (Dobrowski et al., 2011). To accommodate California's ecological diversity, with regions ranging from forested mountain ranges to deserts, we examined local environmental needs by modeling at both state‐wide and state ecoregion scales, using U.S. Environmental Protection Agency (EPA) Level III ecoregion designations. There are 13 Level III ecoregions in California (Table S1.1; Griffith et al., 2016). Although Level IV Ecoregions subdivide the state into finer‐scale habitat regions, Level III is appropriate for this study as there are not enough occurrences per Level IV Ecoregion to build species distribution models.

Species occurrence data used in this study were from a statewide survey of *C. townsendii* in California conducted by Harris et al. (2019). Briefly, methods included field surveys from 2014 to 2017 incorporating both historical roost sites, and a stratified random sampling scheme. Sample sites were selected from a grid of randomly numbered 10 × 10 km cells, stratified by EPA Level III ecoregions to ensure representation of California's disparate habitat types. Areas where *C. townsendii* is not known to occur (e.g., urban core, highest elevations in the Sierra) were excluded. Sites systematically excluded from survey include topographical features inaccessible to surveyors (e.g., cliffs; steep, technical terrain, and underground workings deemed unsafe to enter). Also excluded were areas where survey targets could not be identified during the desktop review process (e.g., landscapes that did not have recognizable abandoned buildings, bridges, or cavern‐like rock formations present). Notably, Ecoregion 7 (Central California Valley) lacks desktop identifiable roost features and historical occurrence records, and is predominantly on private land, resulting in this ecoregion having limited representation in the original survey effort. Occurrence records from the Global Biodiversity Information Facility (https://www.gbif.org) also show a lack of historic or recent occurrence of *C. townsendii* in Ecoregion 7. The lack of detections in this ecoregion likely reflects the reality of a roost‐limited, high disturbance habitat, but also may reflect a climatically unsuitable habitat for *C. townsendii*. Therefore, the limited representation of Ecoregion 7 in the original survey effort likely does not dramatically influence the model results.

Some degree of convenience bias is also present in the data, though not due to a priori sample exclusion criteria. While public property and distance from roads were not selection criteria for survey visits, recognition of potential roost features, and accessibility of such features to survey were far more likely on public jurisdictions than on private land. Similarly, given resource constraints, cells were more likely to be selected for if they contained several potential roost features and were within day‐trip hiking distance, or were adjacent to grids with existing detections. While numerous remote sites were sampled in the original survey effort, the prioritization of historical/known roost sites, coupled with feasibility constraints, introduces some degree of systematic bias against *C. townsendii* detections at greatest distance from roads.

**FIGURE 1:**
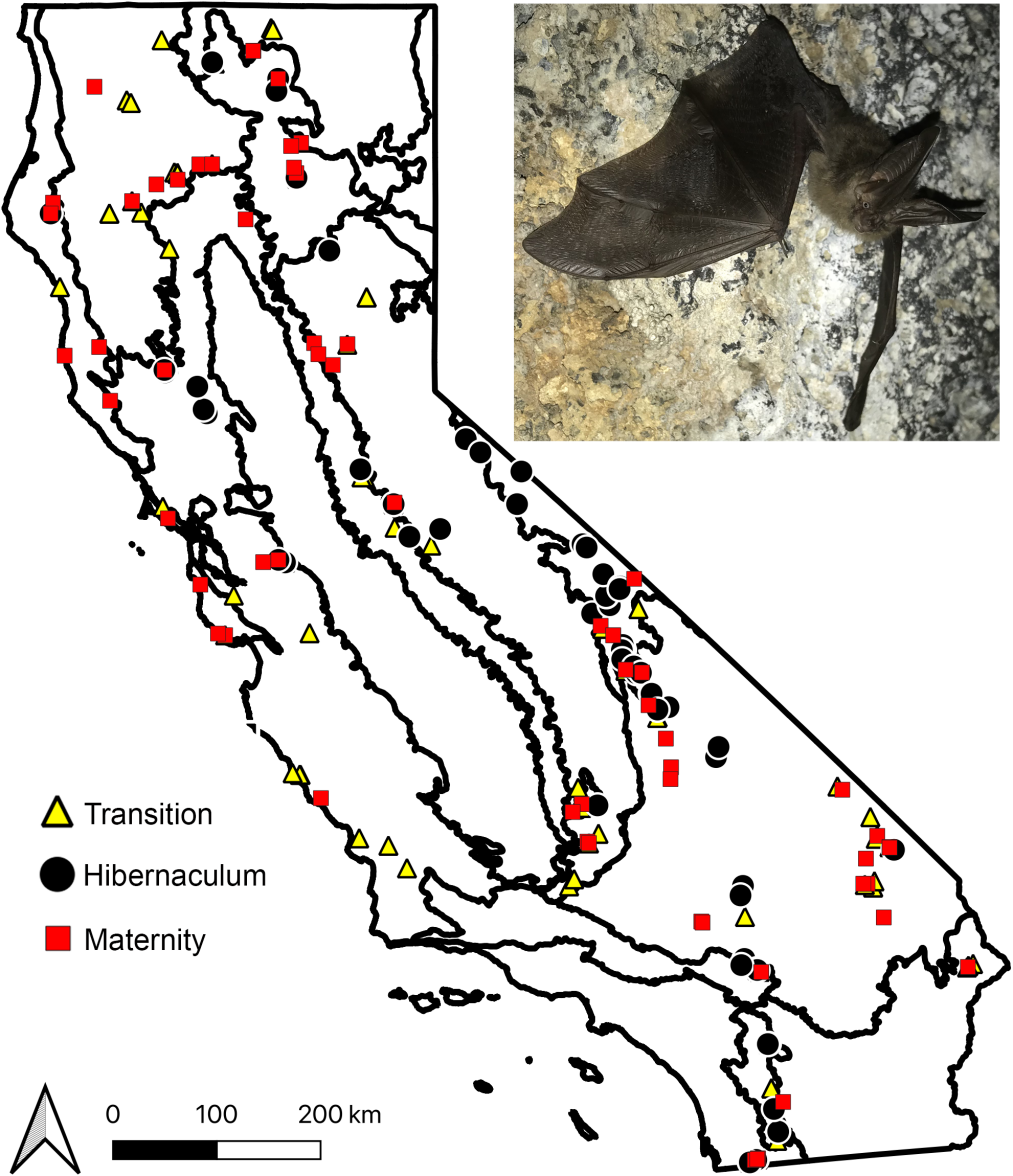
Map of recorded roosts of *Corynorhinus townsendii* in California from Harris et al. (2019) survey efforts. Image of *C. townsendii* provided by Devaughn Fraser.

*Corynorhinus townsendii* presence at roost sites was based on visual bat sightings. From these survey efforts, we have visual occurrence data for 65 maternity roosts, 82 hibernation roosts (hibernacula), and 91 active‐season non‐maternity roosts (transition roosts) for a total of 238 occurrence records (Figure 1, Table S1.1).

### 2.2 Ecogeographical factors

We downloaded climatic variables from WorldClim 2.1 bioclimatic variables (Fick & Hijmans, 2017) at a resolution of five arcmin for broad‐scale analysis and 30 arcsec for our ecoregion‐specific analyses. To calculate elevation and slope, we used a digital elevation model (U.S. Geological Survey, 2019) in ArcGIS 10.8.1 (ESRI, Redlands, California, USA). The chosen set of environmental variables reflects knowledge on climatic conditions and habitat relevant to bat physiology, phenology, and life history (Ancillotto et al., 2016; Loeb & Winters, 2013; Razgour, 2015; Razgour et al., 2011; Rebelo et al., 2010). To trim the global environmental variables to the same extent (the state of California), we used the R package “raster” (Hijmans et al., 2015). We performed a correlation analysis on the raster layers using the “layerStats” function and removed variables with a Pearson's coefficient > 0.7 (see Table 1 for final model variables). Notably, in our study area, elevation was highly correlated with annual temperature (bioclimatic variable 1). We retained elevation for our final models as this variable has been found to be important predictors of roost selection in previous studies of *C. townsendii* (Harris et al., 2019; McClure et al., 2021, 2022; Sherwin et al., 2000). For future climate conditions, we selected three general circulation models (GCMs) based on previous species distribution models of temperate bat species (Razgour et al., 2019) [Hadley Centre Global Environment Model version 2 Earth Systems model (HadGEM3‐GC31_LL; Webb, 2019), Institut Pierre‐Simon Laplace Coupled Model 6th Assessment Low Resolution (IPSL‐CM6A‐LR; Boucher et al., 2018), and Max Planck Institute for Meteorology Earth System Model Low Resolution (MPI‐ESM1‐2‐LR; Brovkin et al., 2019)] and two contrasting greenhouse concentration trajectories (Shared Socio‐economic Pathways; SSPs): a steady decline pathway with CO_2_ concentrations of 360 ppmv (SSP1‐2.6) and an increasing pathway with CO_2_ reaching around 2000 ppmv (SSP5‐8.5; Masson‐Delmotte et al., 2021). We modeled distribution for present conditions future (2061–2080) time periods. Because one aim of our study was to determine the consequences of changing climate, we changed only the climatic data when projecting future distributions, while keeping the other variables constant over time (elevation, slope).

**TABLE 1:** Environmental variable layers included in our species distribution models for *Corynorhinus townsendii* in California.

Variable name	Source	Description
Bio03	WorldClim Version 2.1	Isothermality (°C)
Bio12	WorldClim Version 2.1	Annual precipitation (mm)
Bio15	WorldClim Version 2.1	Precipitation seasonality (Coefficient of variation)
Bio18	WorldClim Version 2.1	Precipitation of warmest quarter (mm)
Slope	USGS	Slope from digital elevation model (^o^)
DEM	USGS	Elevation (m)

### 2.3 Species distribution modeling

We generated distribution maps for total occurrences (maternity + hibernacula + transition, hereafter defined as “combined models”), maternity colonies, hibernacula, and transition roosts. To estimate the present and future habitat suitability for *C. townsendii* in California, we used the maximum entropy (MaxEnt) algorithm in the “dismo” R package (Hijmans & van Etten, 2016) through the advanced computing resources provided by Texas A&M High Performance Research Computing. We chose MaxEnt to aid in the comparisons of state‐wide and ecoregion‐specific models as MaxEnt outperforms other approaches when using small datasets. We created 1000 background points from random points in the environmental layers and performed a fivefold cross‐validation approach, which divided the occurrence records into training (80%) and testing (20%) datasets. We assessed the performance of our models by measuring the area under the receiver operating characteristic curve (AUC; Hanley & McNeil, 1982), where values >0.5 indicate that the model is performing better than random, values 0.5–0.7 indicating poor performance, 0.7–0.9 moderate performance and values of 0.9–1 excellent performance (BCCVL, Hallgren et al., 2016). We also measured the maximum true skill statistic (TSS; Allouche et al., 2006) to assess model performance. The maxTSS ranges from −1 to +1:values <0.4 indicate a model that performs no better than random, 0.4–0.55 indicates poor performance, (0.55–0.7) moderate performance, (0.7–0.85) good performance, and values >0.80 indicate excellent performance (Samadi et al., 2022). Final distribution maps were generated using all occurrence records for each region (rather than the training/testing subset), and the models were projected onto present and future climate conditions. Additionally, because the climatic conditions of the different ecoregions of California vary widely, we generated separate models for each ecoregion in an attempt to capture potential local effects of climate change. A general rule in species distribution modeling is that the occurrence points should be 10 times the number of predictors included in the model, meaning that we would need 60 occurrences in each ecoregion. One common way to overcome this limitation is through the ensemble of small models (ESMs) included in ecospat R package (Breiner et al., 2015, 2018; Di Cola et al., 2017). For our ESMs we implemented MaxEnt modeling, and the final ensemble model was created by averaging individual bivariate models by weighted performance (AUC > 0.5). We also used null model significance testing with to evaluate the performance of our ESMs (Raes & Ter Steege, 2007). To perform null model testing we compared AUC scores from ESMs to the AUC from 100 null models using randomly generated presence locations equal to the number used in the developed distribution model. All ecoregion models outperformed the null expectation (*p* < .002).

### 2.4 Estimating range shifts

For each of the three GCMs and each RCP scenario, we converted the probability distribution map into a binary map (0 = unsuitable, 1 = suitable) using the threshold that maximizes sensitivity and specificity (Liu et al., 2016). To create the final maps for each SSP scenario, we summed the three binary GCM layers and took a consensus approach, meaning climatically suitable areas were pixels where at least two of the three models predicted species presence were retained (Araújo & New, 2007; Piccioli Cappelli et al., 2021). We combined the future binary maps (*fmap*) and the present binary maps (*pmap*) following the formula *fmap* × 2 + *pmap* (from Huang et al., 2017) to produce maps with values of 0 (areas not suitable), 1 (areas that are suitable in the present but not the future), 2 (areas that are not suitable in the present but suitable in the future), and 3 (areas currently suitable that will remain suitable) using the raster calculator function in QGIS. We then calculated the total area of suitability, area of maintenance, area of expansion, and area of contraction for each binary model using the “BIOMOD_RangeSize” function in R package “biomod2” (Thuiller et al., 2016).

## 3 RESULTS

### 3.1 Current models

#### 3.1.1 State‐wide models

**FIGURE 2:**
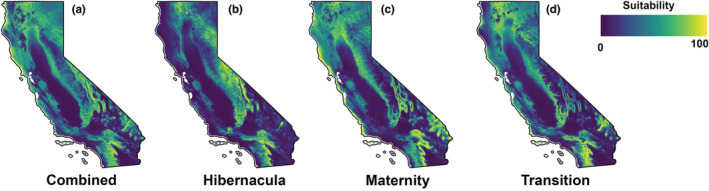
Maps showing the present habitat suitability (probability of occurrence) for *Corynorhinus townsendii* in California based on known roost locations. (a) Model based on all roost occurrence records state‐wide, (b) model based on hibernacula only, (c) model based on maternity colonies only, and (d) model based on active‐season non‐maternity (transition) roosts only. The color ramp corresponds to predicted habitat suitability, where dark blue indicates low habitat suitability and yellow indicates high habitat suitability (scaled 0–100).

Both combined and life‐history‐explicit models showed moderate predictive performance (Table S1.2): combined (AUC = 0.81, MaxTSS = 0.51), hibernacula (AUC = 0.86, MaxTSS = 0.57), Transition (AUC = 0.8, MaxTSS = 9.47), and with maternity models performing the poorest of the three (AUC = 0.78, MaxTSS = 0.43). In the combined and maternity models, precipitation in the warmest quarter provided the highest contribution among environmental variables (Table S1.3). Suitability increased with increasing precipitation in the combined model (S2.1). In the maternity model, the highest suitability is in areas with precipitation in the warmest quarter around 20 mm, with suitability decreasing at higher levels of precipitation (S2.3). For hibernacula, elevation contributed most to the model—suitability generally increases up to 3000 m of elevation, at higher elevation suitability decreases (S2.2). Finally, annual precipitation contributed the most to the transition model, with annual precipitation 250–1200 mm maximizing suitability (S2.4). Areas of suitability for the combined model are distributed across the state, with an area of approximately 150,191 km^2^ (Figure 2a, Table 2), occupying 37% of the land area of California. Suitable areas are found in all ecoregions of the state, excluding the Central California Valley and much of the Sonoran Basin and Range (Ecoregions 7 and 81). The hibernacula model had about 91,503 km^2^ of suitable area in the present, occupying 22% of California (Figure 2b, Table 2). Most notably, coastal areas that are suitable in the combined model are not suitable in the hibernacula‐only model. The maternity model showed the highest suitability throughout the state, distributed across about 162,224 km^2^, or 40% of the state (Figure 2c, Table 2). The transition model predicted an area of approximately 120,002 km^2^ (29%) with areas in the Coast Range, Sierra Nevada region, and the Central Basin and Range (Ecoregions 1, 5, 13) showing lower levels of suitability than the combined model (Figure 2d, Table 2).

**TABLE 2:** Predicted suitable area for *Corynorhinus townsendii* roosts in California under present and future conditions for all temporal and geographic subsets modeled.

Geographic extent/Colony type	Range shift	Present	SSP1‐2.6 2061‐2080	SSP5‐8.5 2061‐2080
All Colonies State‐Wide	Total suitable habitat (km^2^)	150,191	142,245	123,865
	Expansion (km^2^)		2728	33,530
	Contraction (km^2^)		10,647	7205
	Maintained (km^2^)		139,544	116,661
	Change compared to current distribution (km^2^, %)		−7945 (−5%)	−26,325 (−17.5%)
Hibernacula State‐Wide	Total suitable habitat (km^2^)	91,503	87,538	70,081
	Expansion (km^2^)		3931	7424
	Contraction (km^2^)		7897	28,846
	Maintained (km^2^)		83,607	62,658
	Change compared to current distribution (km^2^, %)		−3966 (−4%)	−21, 422 (−23%)
Maternity State‐Wide	Total suitable habitat (km^2^)	162,224	142,460	130,307
	Expansion (km^2^)		3428	9445
	Contraction (km^2^)		23,192	41,362
	Maintained (km^2^)		139,033	120,862
	Change compared to current distribution (km^2^, %)		−19,764 (−12%)	−31,918 (−20%)
Transition State‐Wide	Total suitable habitat (km^2^)	120,002	123,287	125,626
	Expansion (km^2^)		7729	22,455
	Contraction (km^2^)		4444	16,831
	Maintained (km^2^)		115,558	103,171
	Change compared to current distribution (km^2^, %)		+3286 (+3%)	5624 (+5%)
Ecoregion 1	Total suitable habitat (km^2^)	6979	7009	6804
	Expansion (km^2^)		320	271
	Contraction (km^2^)		289	446
	Maintained (km^2^)		6690	6533
	Change compared to current distribution (km^2^, %)		−31 (+0.4%)	−175 (−2.5%)
Ecoregion 4	Total suitable habitat (km^2^)	3128	2812	3233
	Expansion (km^2^)		283	999
	Contraction (km^2^)		599	894
	Maintained		2529	2234
	Change compared to current distribution (km^2^, %)		−316 (−10%)	+105 (+3%)
Ecoregion 5	Total suitable habitat (km^2^)	22,675	18,689	12,035
	Expansion (km^2^)		1040	375
	Contraction (km^2^)		5027	11,016
	Maintained (km^2^)		17,648	11,660
	Change compared to current distribution (km^2^, %)		−3987 (−18%)	−10,641 (−47%)
Ecoregion 6	Total suitable habitat (km^2^)	54,439	12,978	27,417
	Expansion (km^2^)		0.5	302
	Contraction (km^2^)		41,462	302
	Maintained (km^2^)		12,978	27,115
	Change compared to current distribution (km^2^, %)		−41,462 (−76%)	−27,022(−59%)
Ecoregion 8	Total suitable habitat (km^2^)	4337	4000	4292
	Expansion (km^2^)		127	356
	Contraction (km^2^)		464	401
	Maintained (km^2^)		3873	3936
	Change compared to current distribution (km^2^, %)		−337 (−8%)	−45 (−1%)
Ecoregion 13	Total suitable habitat (km^2^)	8977	8272	7679
	Expansion (km^2^)		186	269
	Contraction (km^2^)		828	1567
	Maintained (km^2^)		8086	7410
	Change compared to current distribution (km^2^, %)		−705 (−8%)	−1298 (−14%)
Ecoregion 14	Total suitable habitat (km^2^)	24,028	7912	9682
	Expansion (km^2^)		0	0
	Contraction (km^2^)		16,116	14,347
	Maintained (km^2^)		7912	9682
	Change compared to current distribution (km^2^, %)		−16,116 (−67%)	−14,347 (−60%)
Ecoregion 78	Total Occupied Area (km^2^)	11,857	10,669	12,111
	Expansion (km^2^)		1637	2975
	Contraction (km^2^)		2825	2721
	Maintained (km^2^)		9032	9136
	Change Compared to current distribution (km^2^, %)		−1187 (−10%)	+254 (+2%)
Ecoregion 85	Total suitable habitat (km^2^)	4556	3852	4719
	Expansion (km^2^)		17	11
	Contraction (km^2^)		722	148
	Maintained (km^2^)		3834	4408
	Change compared to current distribution (km^2^, %)		−704 (−15%)	+163 (+4%)

#### 3.1.2 Ecoregion‐specific models

**FIGURE 3:**
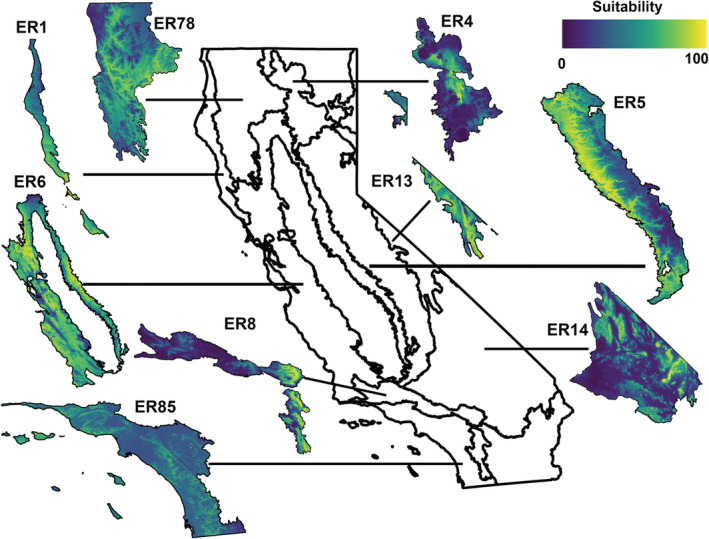
Map showing level III ecoregion‐specific habitat suitability for *Corynorhinus townsendii* in California based on known roost locations. The color ramp corresponds to predicted habitat suitability, where dark blue indicates low habitat suitability and yellow indicates high habitat suitability.

The predictive performance from each of our ecoregion models were all acceptable with respect to AUC (Table S1.2), with excellent performance for six ecoregions and moderate performance for three ecoregions. Additional details on model performance and contribution of environmental variables to each model are reported in Tables S1.2 and S1.3. The ecoregion‐specific models for Ecoregions 1 and 85 had different distributions of suitable area than the respective regions in the combined model (Figure 3). Ecoregions 4 and 8 showed substantially less suitable area when compared with the same area in the combined model. Ecoregion 5 generally matched the pattern of distribution in the combined model, but the ecoregion model had more areas of high suitability in the eastern part of the region. The area of suitability in the individual model for Ecoregion 6 is also generally similar to the same area in the combined model but has less suitable area in the most northern part of the ecoregion, and occupied areas in the rest of the region have higher suitability. When compared to the combined model, Ecoregion 13 has similar areas of high suitability except in the north. Ecoregion 14 had similar a similar distribution, but lower suitability when compared to the combined model. In general, the ecoregion‐specific models showed similar patterns of suitability to, but with better model performance than the combined model. Variable contribution to each ecoregion model can be found in Table S1.3. Overall, precipitation (annual precipitation, precipitation of the warmest quarter, or precipitation seasonality) was the highest contributing variable in five of the ecoregion models (Ecoregions 1, 5, 6, 14, and 78). Suitability was maximized at precipitation of around 20–30 mm in Ecoregions 1,5, and 6 (Figures S2.5, S2.7, S2.8). In Ecoregion 14, suitability increased with increasing precipitation (S2.9). The remaining ecoregions were best explained by elevation and isothermality (Ecoregions 13 and 85 and Ecoregions 4 and 8, respectively; Table S1.3). In Ecoregion 13, suitability generally increased with increasing elevation, maximizing around 2000–2500 m (S2.10). In Ecoregion 85 however, suitability decreases with increasing elevation (S2.11).

### 3.2 Changes in distribution

Six maps of habitat suitability (3 GCMs × 2 SSPs) were generated for each geographic category. Binary maps created using the threshold as stated above were used to assess areas of expansion and contraction.

#### 3.2.1 State‐wide models

**FIGURE 4:**
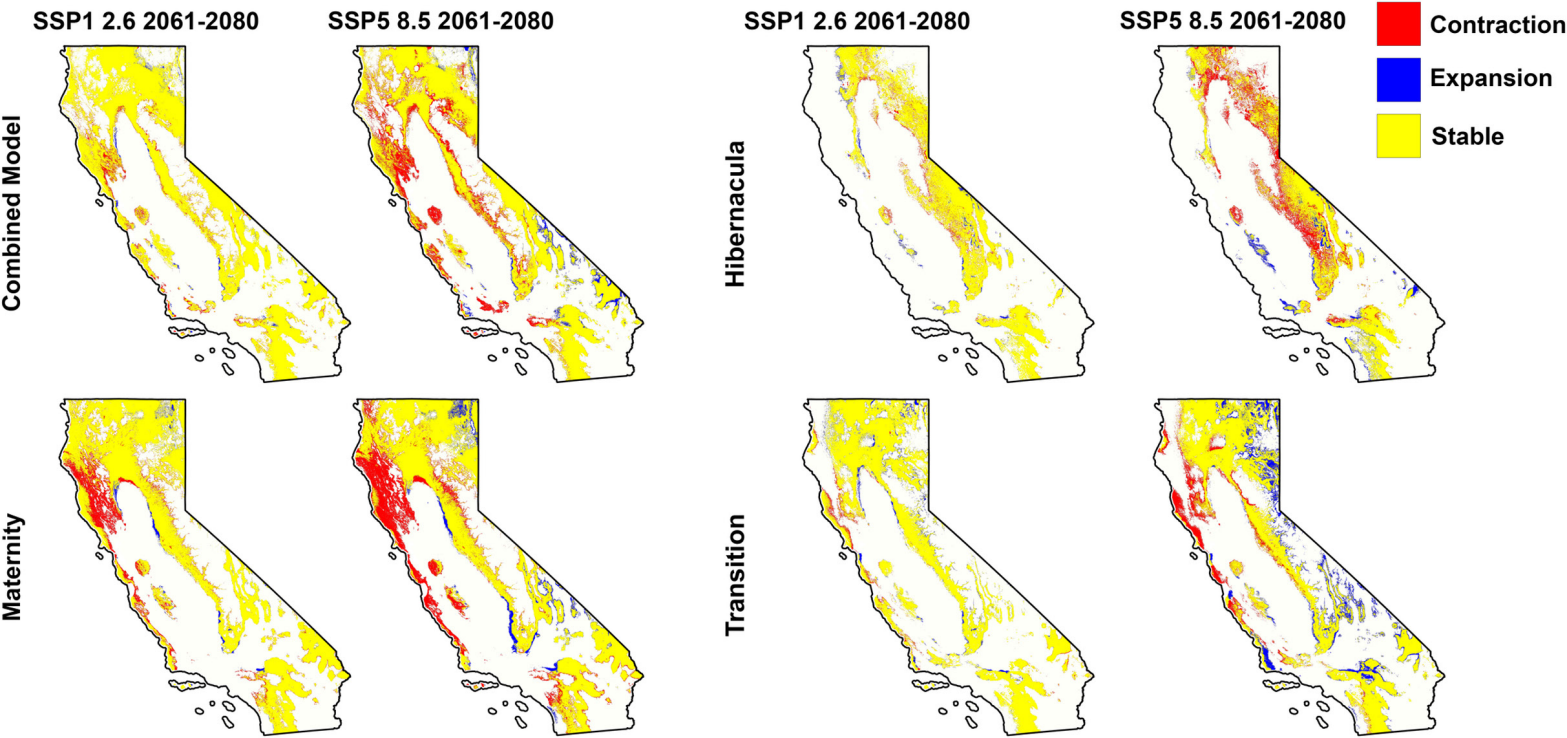
Climate‐related habitat suitability shifts in extent and location in *Corynorhinus townsendii* in California based on known roost locations. Colors indicate areas of contraction (red), expansion (blue), and areas that are currently suitable that will remain suitable in the future (yellow).

The total change in suitable areas for *C. townsendii* across the state are reported in Table 2. The combined, hibernacula, and maternity model approaches predict an overall contraction of suitable area for *C. townsendii* between 2061 and 2080, with larger reductions in the SSP5‐8.5 scenario. Reduction in the suitable habitat of the combined model was predicted to occur in most of the ecoregions, however, some expansion was predicted in eastern areas of the state, within the Mojave Basin and Range (Figure 4). In the hibernacula model, reduction in suitable area is predicted to occur along areas currently suitable, with an expansion in western areas, within the coastal mountains. Suitability for maternity colonies is expected to decrease largely in western areas of the state. The area of suitable habitat for transition colonies is expected to increase under both climate change scenarios, with a larger increase seen in SSP5‐8.5. Under this scenario, suitable habitat on the coast is expected to decrease, while areas in eastern California are expected to increase in suitability, corresponding to an overall range shift.

#### 3.2.2 Ecoregion models

**FIGURE 5:**
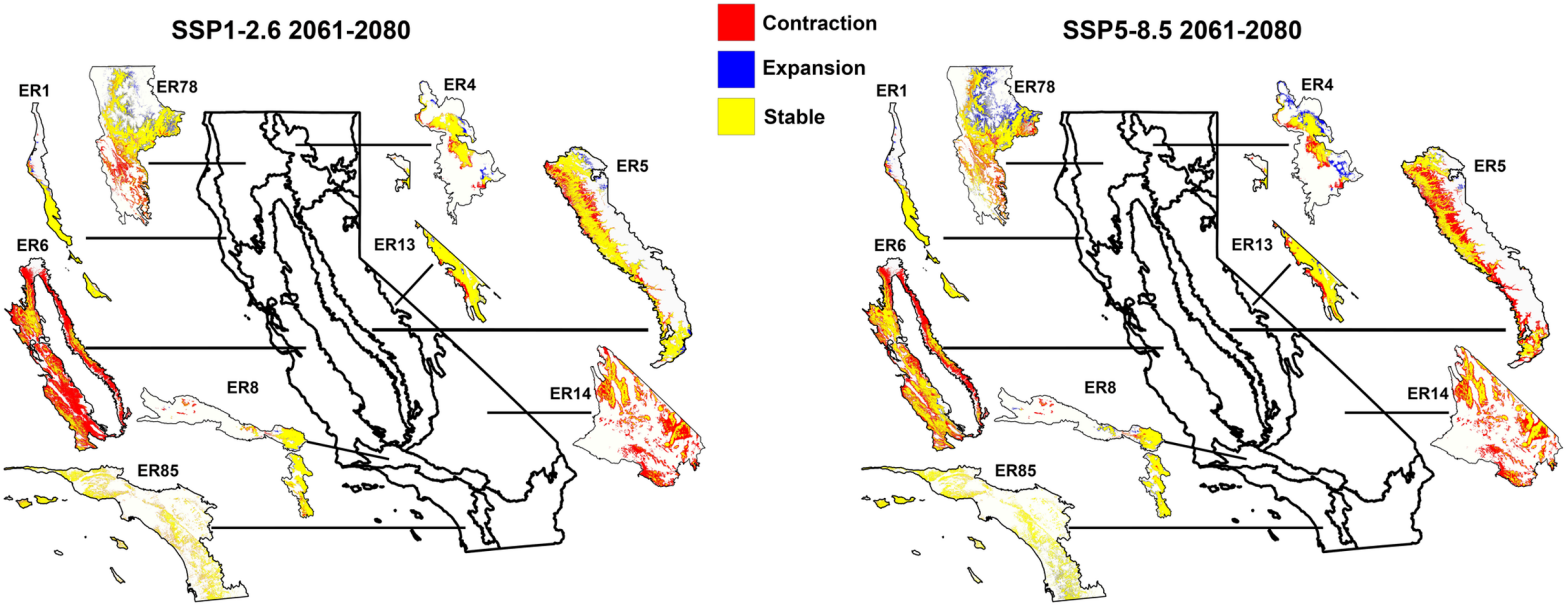
Climate‐related habitat suitability shifts for level III ecoregion‐specific models of *Corynorhinus townsendii* in California based on known roost locations. Colors indicate areas of contraction (red), expansion (blue), and areas that are currently suitable that will remain suitable in the future (yellow).

Details of all changes in suitability per ecoregion under each RCP scenario and time period are reported in Table 2. Five of the ecoregions are projected to decrease in suitable area under both SSP scenarios and time periods (Figure 5, Table 2). Ecoregion 6 is predicted to lose the highest percentage of its range (59–76%). Ecoregion 14 is also predicted to lose suitability in a significant proportion of its current range in California (60–67%). Under the SSP1‐2.6 scenario, Ecoregion 1 is expected to have a slight increase in suitable habitat and in the SSP5‐8.5 scenario Ecoregions 4, 78, and are 85 projected to have an increase in suitable area.

## 4 DISCUSSION

We estimated the most important environmental factors influencing the distribution of *C. townsendii* colonies in California and provided seasonal and regional projections of suitable habitat under present conditions and in response to simulated lower and higher future concentrations of global greenhouse gases. While seasonal, life‐history‐explicit species distribution models are recognized as important for taxa that have long‐distance migration, taxa with small‐scale seasonal movements have largely been neglected in modeling studies (Smeraldo et al., 2018). Our study highlights how ecological need differences between phenological stages contribute to differences in seasonal distribution of *C. townsendii*. Additionally, by modeling each ecoregion separately, our study highlights how environmental needs vary across the species' range in California.

Overall, our results indicate that environmental factors driving *C. townsendii* distribution differ across temporal and geographic scales, but precipitation is the most important factor predicting *C. townsendii* presence in the majority of our models. Availability of water, in terms of either distance to permanent water sources or amount of precipitation, is important for insectivorous bats for both foraging and drinking water and the association between maternity colonies and annual precipitation is to be expected, as female insectivorous bats also have a significant increase in water needs during pregnancy and lactation (Adams & Hayes, 2008; Rainho & Palmeirim, 2011). Precipitation is also an important factor to hibernacula as it contributes to cave humidity (Perry, 2013). High humidity within hibernacula reduces evaporative water loss during hibernation (Speakman & Thomas, 2003). Our results support precipitation as an important factor for this species at multiple life phases. Elevation is another important variable contributing to the hibernacula models, with suitability increasing with elevation up to ~2750 m, consistent with previous studies of *C. townsendii* reporting the species hibernating at >1500 m (Gillies et al., 2014; Harris et al., 2019; Hayes et al., 2011; Szewczak et al., 1998; Whiting et al., 2021). However, recent modeling approaches predict higher suitability for *C. townsendii* hibernacula at elevations <1000 m. Differences in predicted elevation are likely due to differences in occurrence data, environmental predictor variables, study extent (including latitude), and modeling techniques used. Higher elevation is linked to lower temperature, which is important for hibernating bats as they must select sites with stable, low temperatures to ensure that their metabolic rate will not exhaust fat reserves before hibernation ends (Humphrey, 1978; Perry, 2013; Thomas et al., 1990). As our initial model building found a high correlation between temperature and elevation in our study area, annual temperature is potentially also driving the distribution of high suitability in the hibernacula models.

The differences in environmental needs are reflected in the differences in the distribution of predicted suitable habitat between our models. The predicted distribution of suitable habitat for *C. townsendii* roosts differed between the combined, hibernacula, maternity, and transition models, consistent with recent work examining temporally explicit distribution models in locally migratory bats (Smeraldo et al., 2018). The combined, maternity, and transition models predict areas of high suitability across the state (Figure 3). On the contrary, the hibernacula model shows less area of high suitability, corresponding to areas of sufficient elevation (Figure 3). Because hibernacula have a more restricted area of suitability in California, the hibernacula model failed to predict the extent of the known range of *C. townsendii* in the state. This highlights how the combined model (encompassing all phenological stages) best captures the broad distribution of *C. townsendii* in California. It also highlights how life‐history‐explicit models are more useful than the combined in describing seasonal niches of *C. townsendii* and could therefore be more useful when planning seasonal survey efforts.

When looking at the ecoregion‐scale models, we see that the environmental variable contributing most to the suitability of *C. townsendii* varies across ecoregions (Table S1.3). However, most ecoregions were best explained by amount of annual precipitation or precipitation in the warmest quarter, consistent with the state‐wide models and previously mentioned physiological needs of temperate insectivorous bats. Isothermality (thermal stability) contributes most to the suitability for two ecoregions (Ecoregions 4 and 8) that have high annual precipitation across the region when compared with other ecoregions. Isothermality is also linked to physiological performance and favorable reproductive outcomes in temperate bat species (Ancillotto et al., 2018). Distribution of suitable area in the ecoregion‐specific models also was largely consistent with the distribution seen in the combined state‐wide model. Overall, these results indicate a large congruence between state‐wide and ecoregion‐specific models, suggesting a state‐wide model is useful in predicting the current distribution of *C. townsendii* in California.

Current global emissions are most consistent with the SSP5‐8.5 scenario (Schwalm et al., 2020). Under the SSP5‐8.5 scenario, model results indicate that the area suitable for *C. townsendii* in California will decrease when considering the state‐wide and ecoregion models, with an exception of a range shift (and overall expansion of suitable area) in the transition roosts. Because the amount of annual precipitation best explained the majority of models, changes in annual precipitation predicted under SSP5‐8.5 scenarios is likely contributing to the shifts of suitable habitat in *C. townsendii*. Notably, although elevation does not change between present and future scenarios, the amount of suitable area for hibernacula is expected to decrease, suggesting the interaction between elevation and precipitation is driving the distribution of *C. townsendii* hibernacula in California. Additionally, the changes in suitable habitat between the state‐wide and ecoregion‐specific models are largely congruent, with the exception of areas in within Ecoregions 4, 14, and 78. In the state‐wide scenario, Ecoregion 14 is expected to have an increase in suitable area, but in the ecoregion‐specific model, this region is expected to experience a notable contraction in suitable area. In contrast, Ecoregions 4 and 78 are expected to have contractions in the state‐wide scenario but are predicted to have a northward expansion. However, unlike the combined model, isothermality also significantly contributes to the projected suitability for the ecoregion‐specific models for Ecoregions 4, 14, and 78—suggesting that future changes in temperature will have different effects on the distribution of *C. townsendii* within these regions. Differences in the projected range shifts between the state‐wide and ecoregion‐specific models provide evidence that the relationship between distribution and predictive environmental variables is not consistent across the *C. townsendii* range within California. The differences in environmental needs across ecoregions may be driving intraspecific variation in climate tolerances, or local adaptation. Previous work in non‐volant small mammals suggests that ecoregion‐level population classifications are sufficient for capturing intraspecific variation to climate response, but additional work is necessary to determine if *C. townsendii* are in fact locally adapted (Smith et al., 2019). Additionally, expansion of modeling to populations beyond California will allow for the full adaptive potential of the species to be addressed.

For the aims of our work (i.e., to model distributions of roost suitability), we considered only roosting bats as species' presence records. We did not include acoustic record or mist net capture as these types of records do not allow us to characterize type of environmental use (e.g., maternity vs. transition roosts), meaning that these types of landscape detections are likely far less ecologically meaningful than the presence of the presumably more limiting roost locations. Excluding foraging locations may underestimate the realized niche, but roost data have successfully produced roost habitat maps for other temperate bat species (McClure et al., 2021, 2022; Smeraldo et al., 2018). Additionally, we did not include roost‐habitat covariates (e.g., humidity, size) in our models because we do not have adequate dimensional or microclimate data for all subterranean and anthropogenic features in the study area. Distance to mines or caves would also not be informative for our model, as our occurrence points were restricted to cavern‐analog locations. Future models including information on cavern characteristics such as entrance size, internal dimensions, and microclimate can help further refine predictions of *C. townsendii* roosts. Additionally, survey efforts did not include monitoring human activity at each roost, therefore disturbance vulnerability of each roost was not determined. Because this species is sensitive to human disturbance, future research to quantify roost disturbance will be critical to management of this at‐risk species. Abundance data (i.e., size of populations) were also not available for the occurrence data used in this study. Information on the abundance of *C. townsendii* in each ecoregion can improve our vulnerability estimates by allowing us to predict proportional changes to species prevalence within the state (Waldock et al., 2021).

The maps of occurrence probability can help guide future work to survey and monitor California populations of *C. townsendii* and provide a baseline for understanding potential impacts of future climate change. Management applications of these results should consider whether features such as caves, abandoned mines, and appropriate anthropogenic structures are available in areas predicted to be environmentally suitable. Our models provide useful data that can be updated over time to incorporate new climate research, adapt to shifting conservation goals, or respond to other impacts such as land use change. Knowledge on the vulnerability of populations within each ecoregion enables land managers to concentrate resources on protecting potential refugia (areas predicted to remain suitable) in their regions. Protection and enhancement of predicted refugia and promotion of connectivity between present and future suitable areas are ways that species distribution models can be used to focus conservation planning (Piccioli Cappelli et al., 2021) .

## AUTHOR CONTRIBUTIONS

**Joseph M. Szewczak:** Data curation (equal); funding acquisition (equal); writing – review and editing (equal). **Leila S. Harris:** Data curation (lead); writing – review and editing (equal). **Michael L. Morrison:** Conceptualization (equal); funding acquisition (equal); supervision (lead); writing – review and editing (equal). **Natalie M. Hamilton:** Conceptualization (equal); formal analysis (lead); writing – original draft (lead). **Scott D. Osborn:** Data curation (equal); funding acquisition (equal); writing – review and editing (equal).

## CONFLICT OF INTEREST

The authors have no conflict of interest to report.

## Supporting information


Figures S1

Table S1

Table S2

Table S3

## Data Availability

We cannot provide original bat occurrence points used in analyses in the interest of protecting sensitive colony locations from disturbance or vandalism. However, our complete analytical dataset, including predictor variables (removed of location data), environmental layers, and R scripts, are available on Dryad, https://doi.org/10.5061/dryad.4j0zpc8f1.
