# Supplementary material for: Predicting habitat suitability for Townsend's big‐eared bats across California in relation to climate change
Source: Ecol Evol. 2022 Dec 15;12(12):e9641. doi: 10.1002/ece3.9641 (PMC9755818; doi:10.1002/ece3.9641)

Figure S2.1. Response plots for combined model

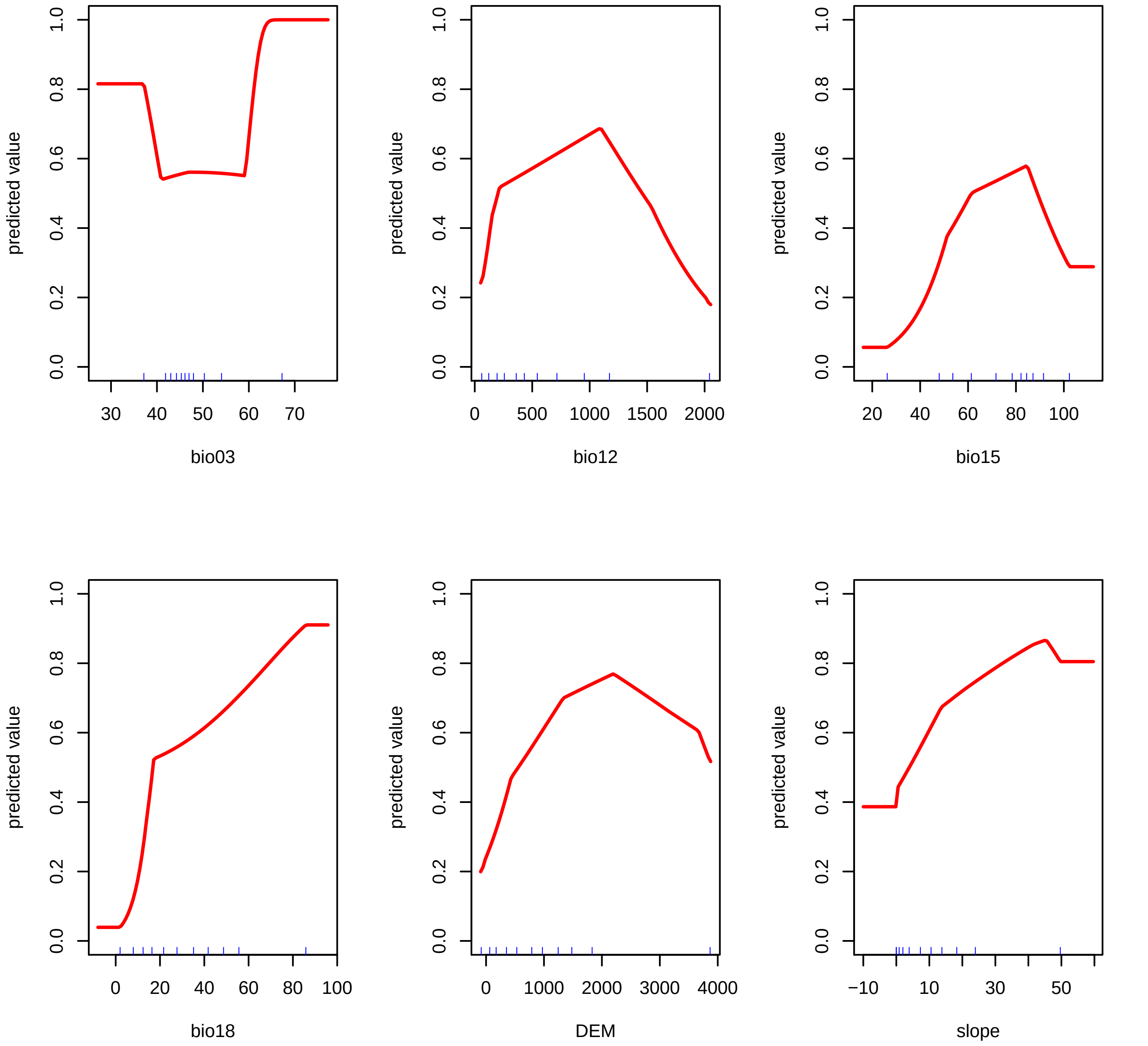

Figure S2.2. Response plots for hibernacula model

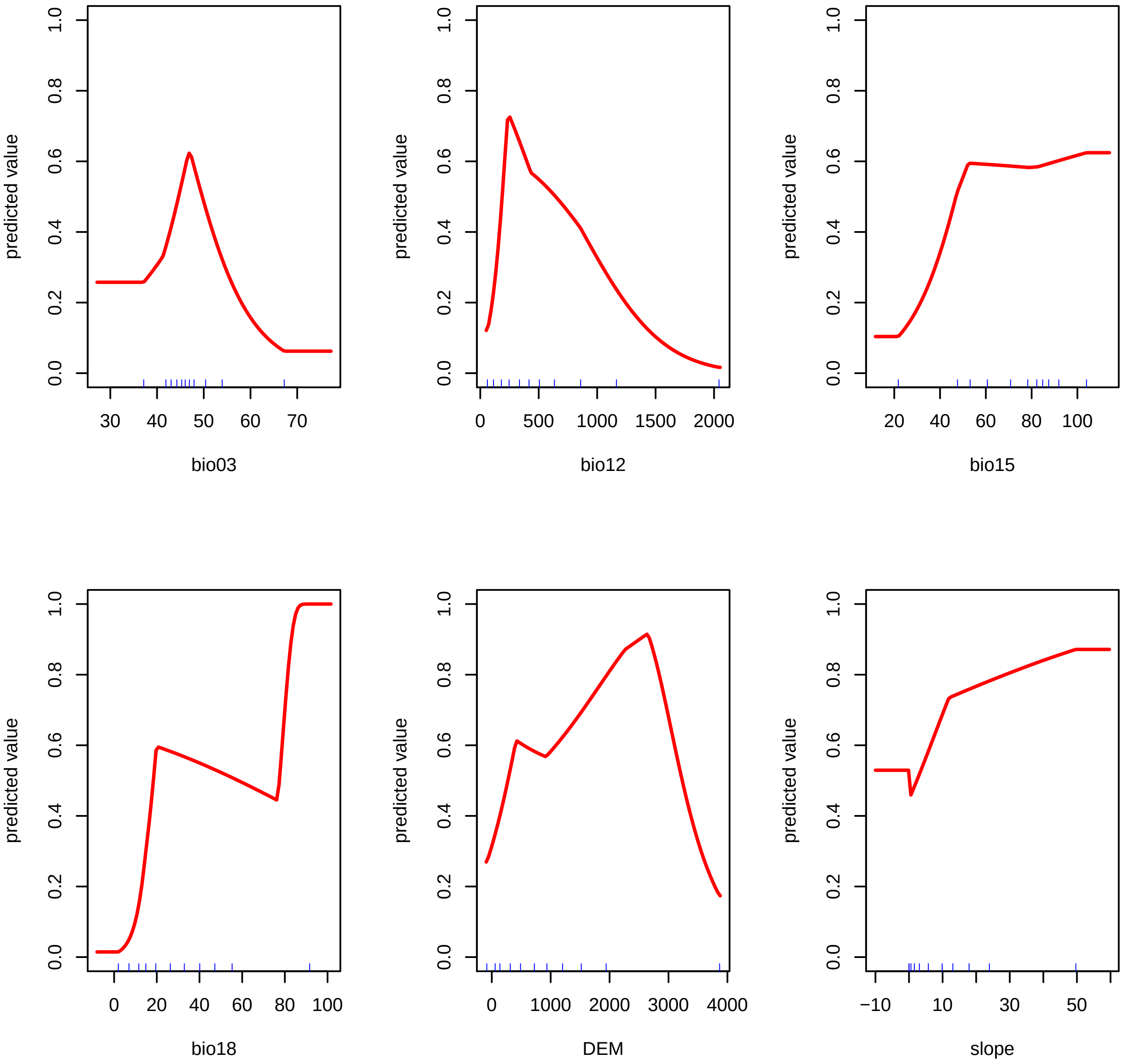

Table S2.3. Response plots for maternity models

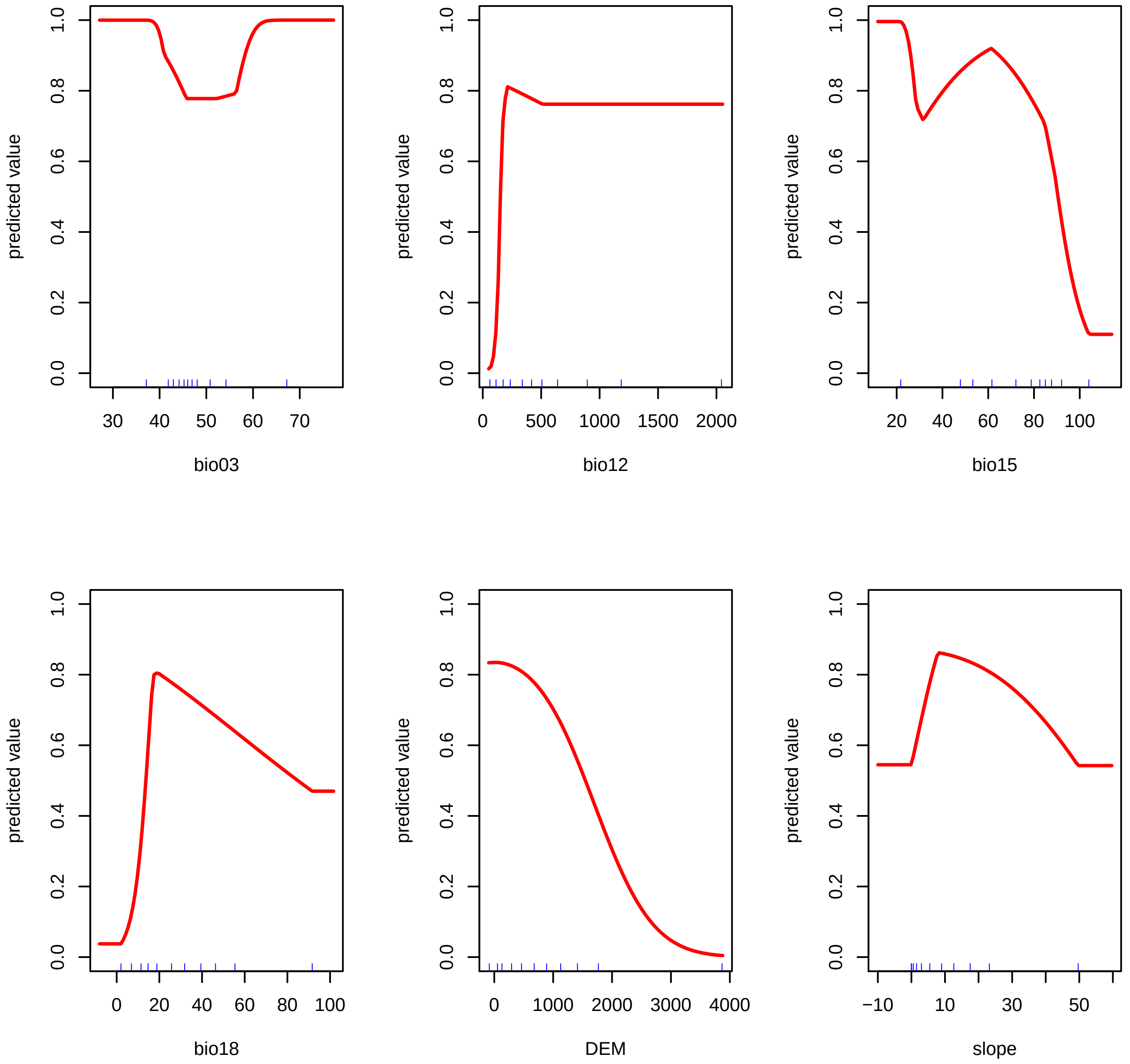

Table S2.4. Response plots for transition model

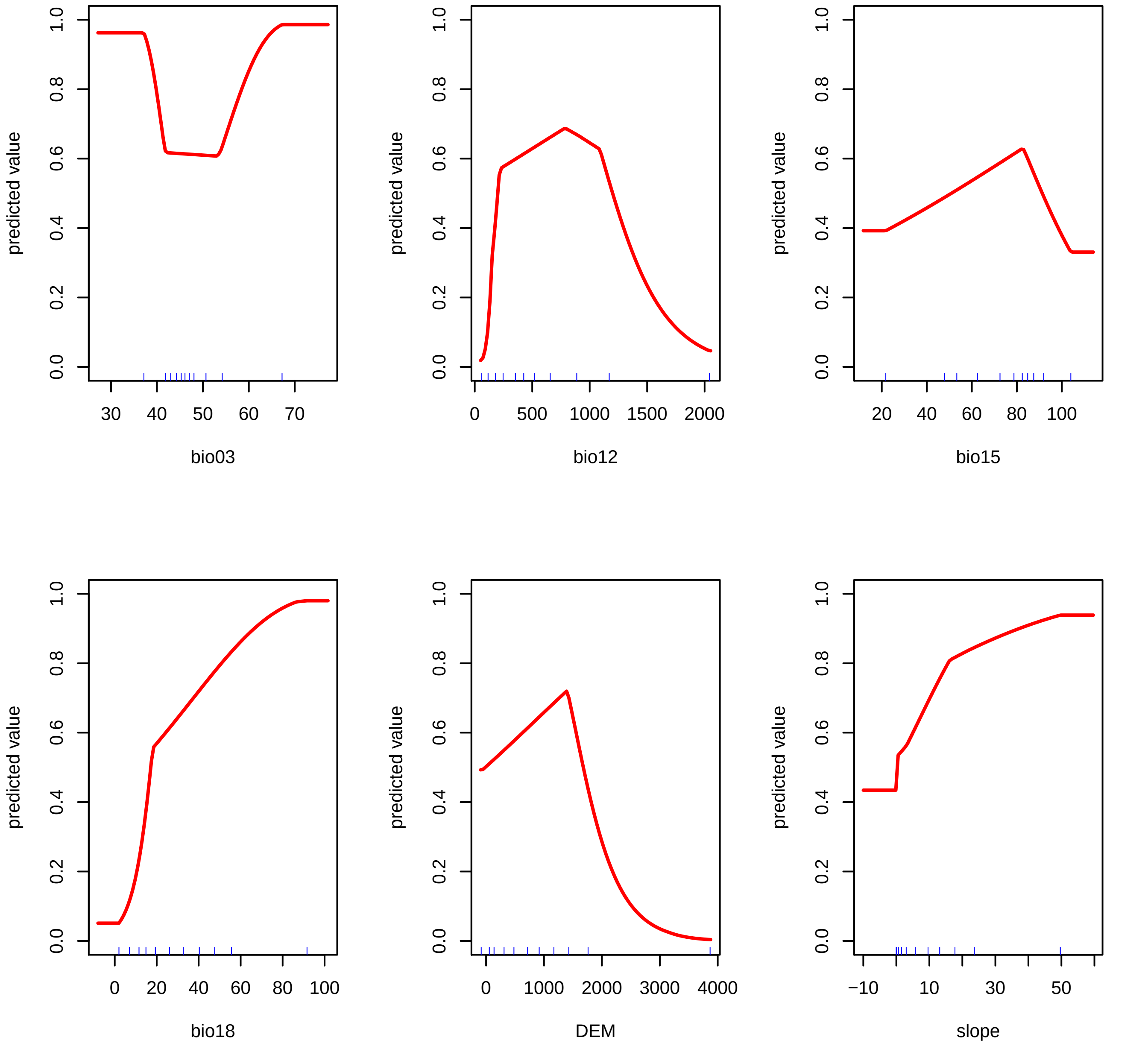

Figure S2.5. Response plot for ecoregion 1

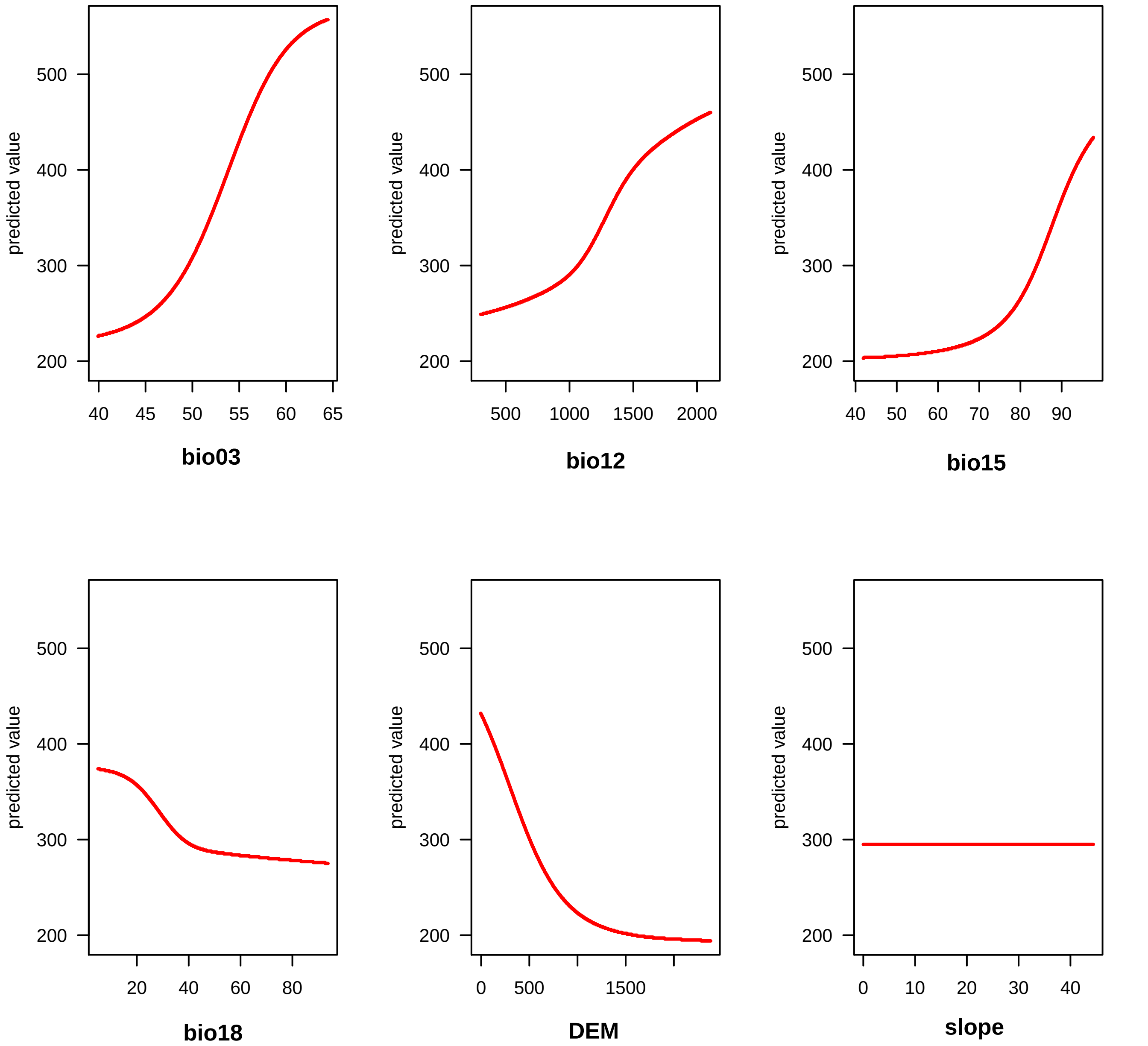

Figure S2.6. Response plots for ecoregion 4

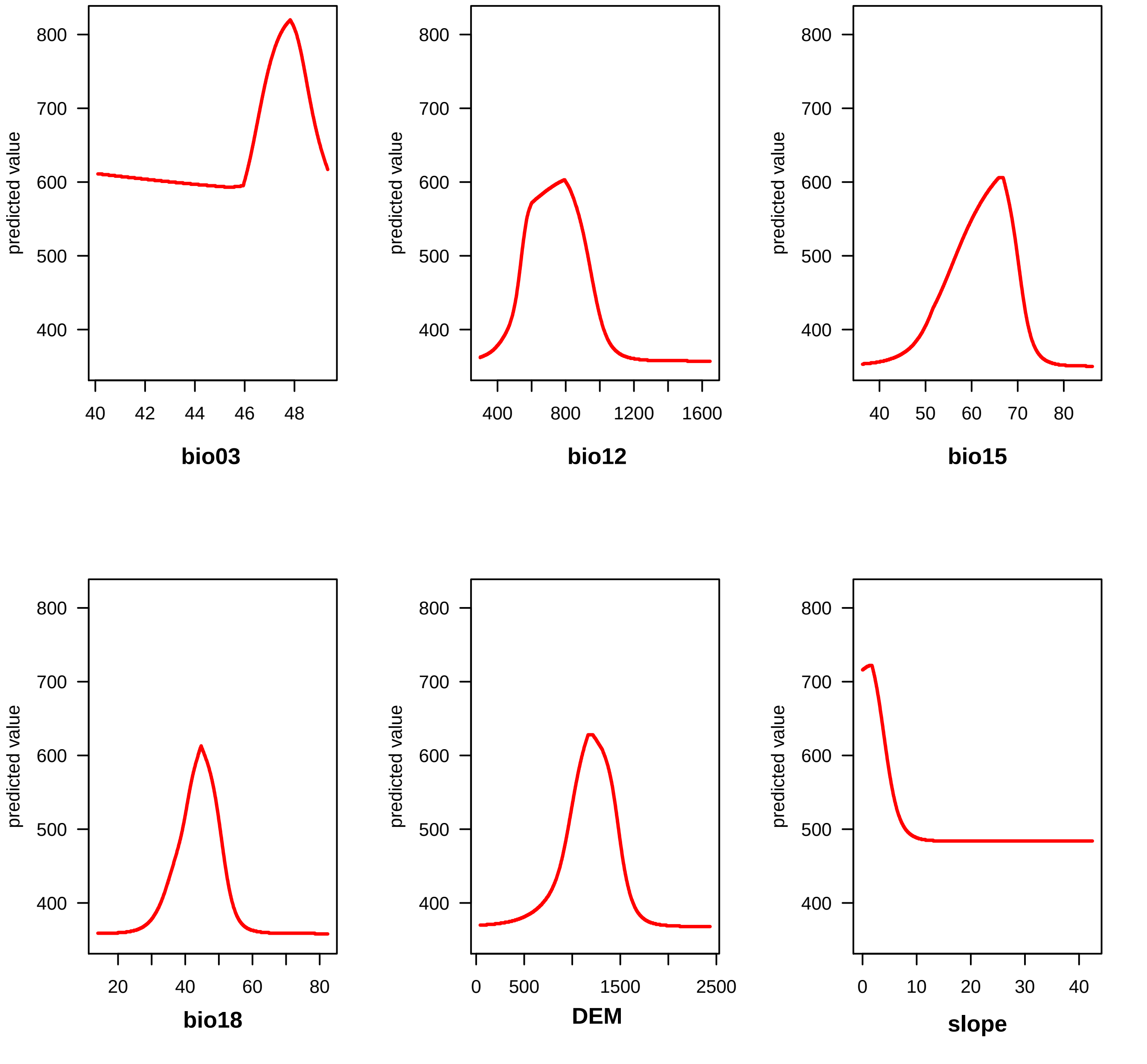

Table S2.7 Response plots for ecoregion 5

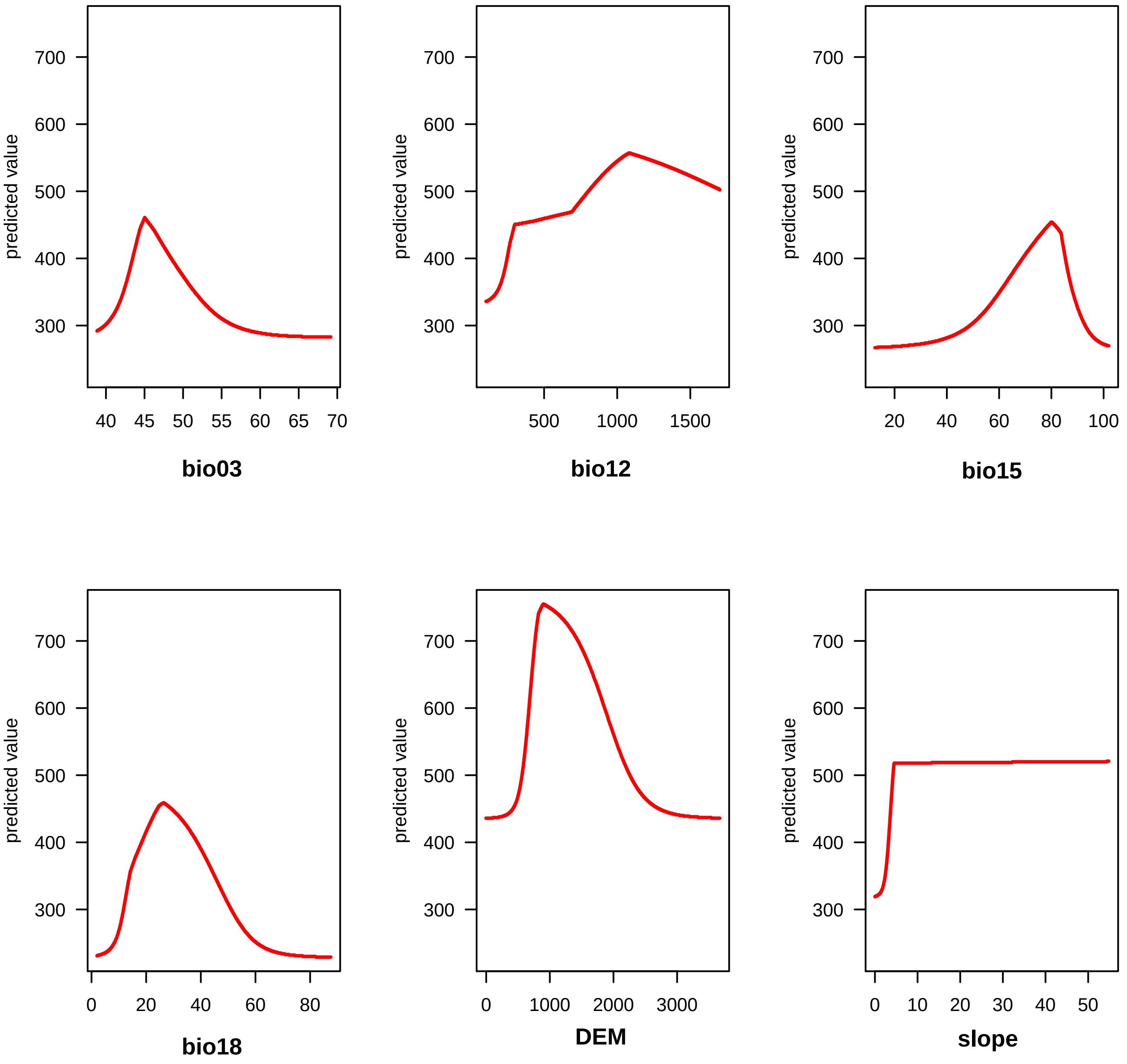

Figure S2.8. Response plots for ecoregion 6

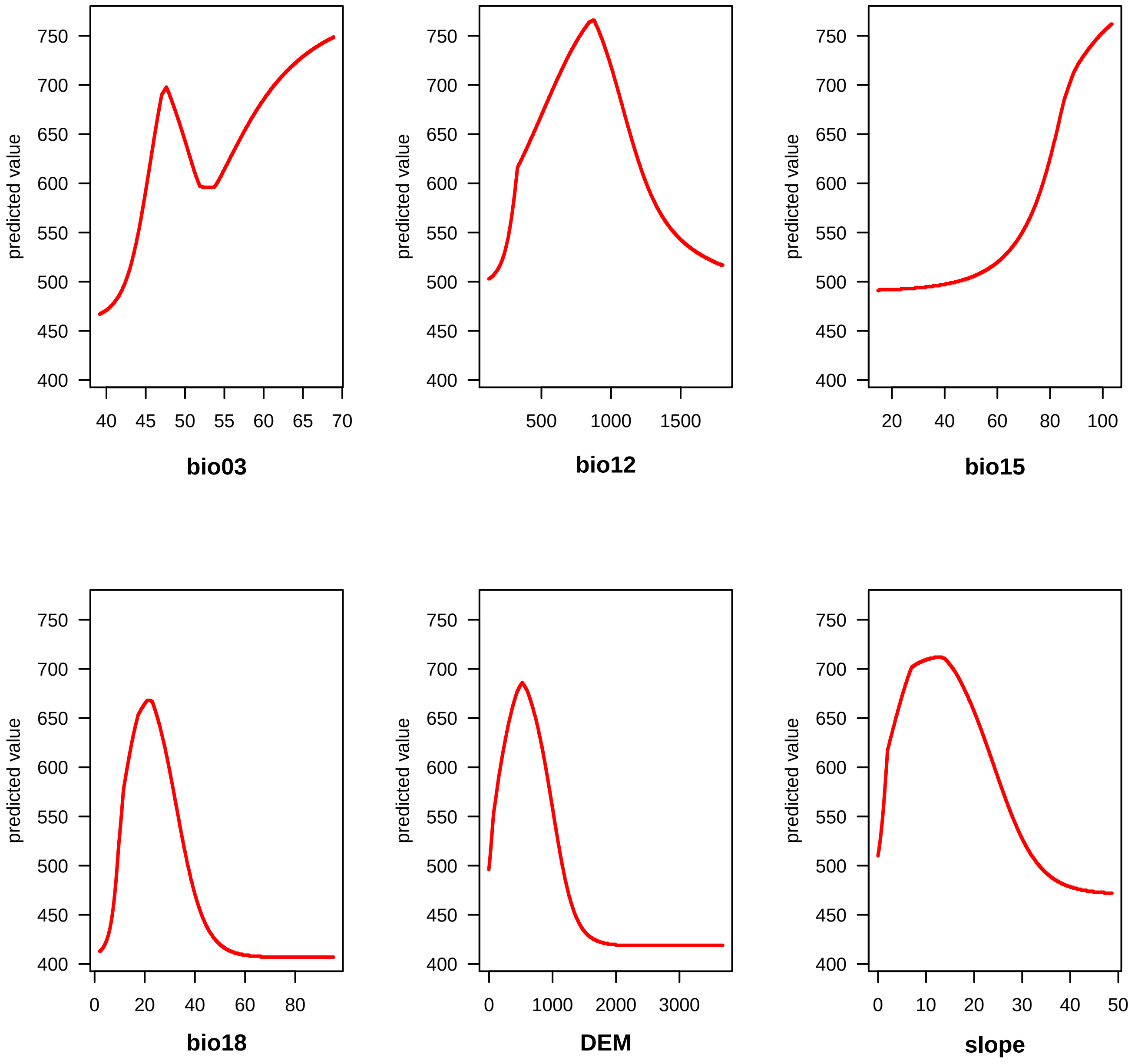

Figure S2.9. Response plots for ecoregion 8

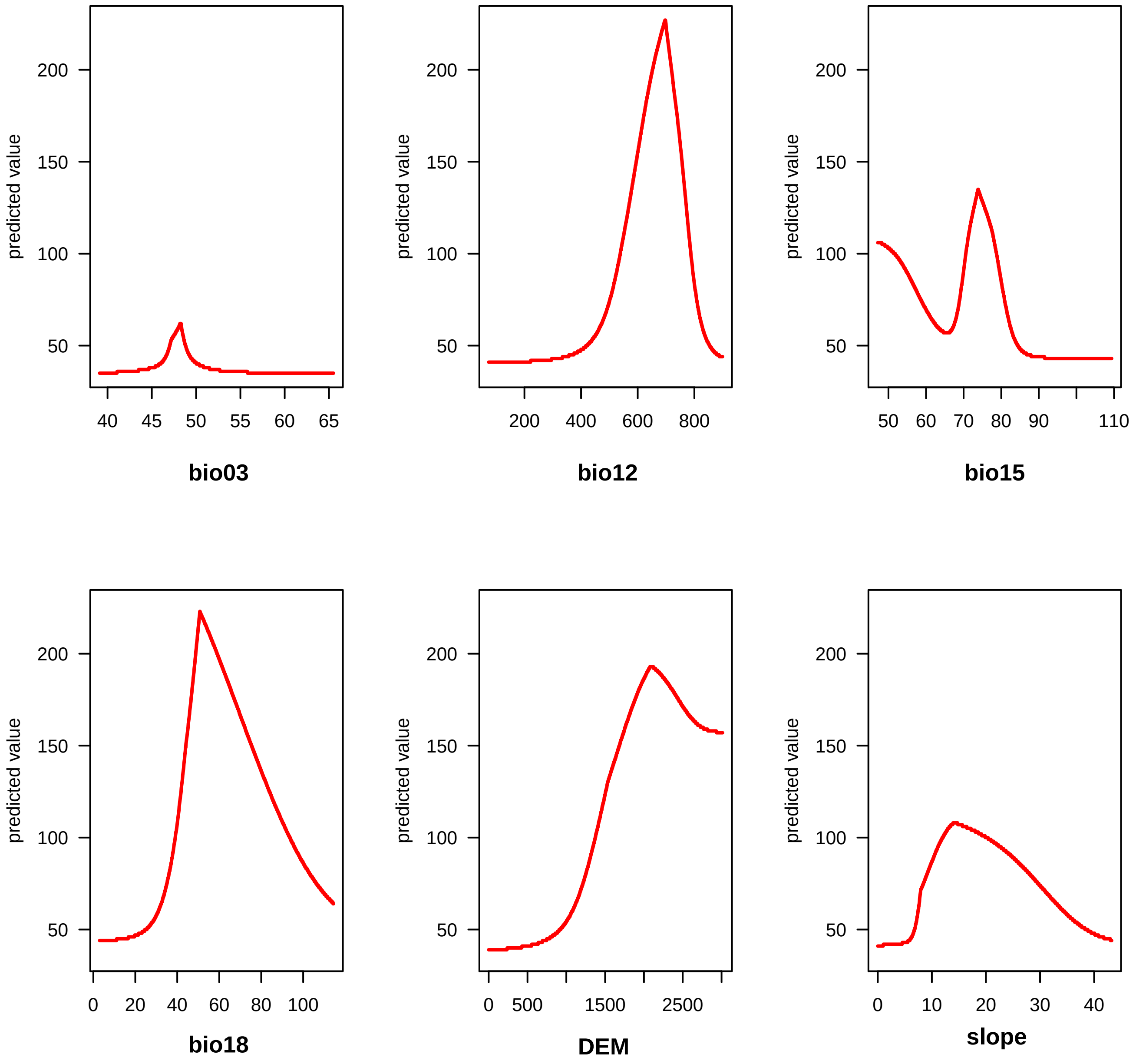

Figure S2.10. Response plots for ecoregion 13

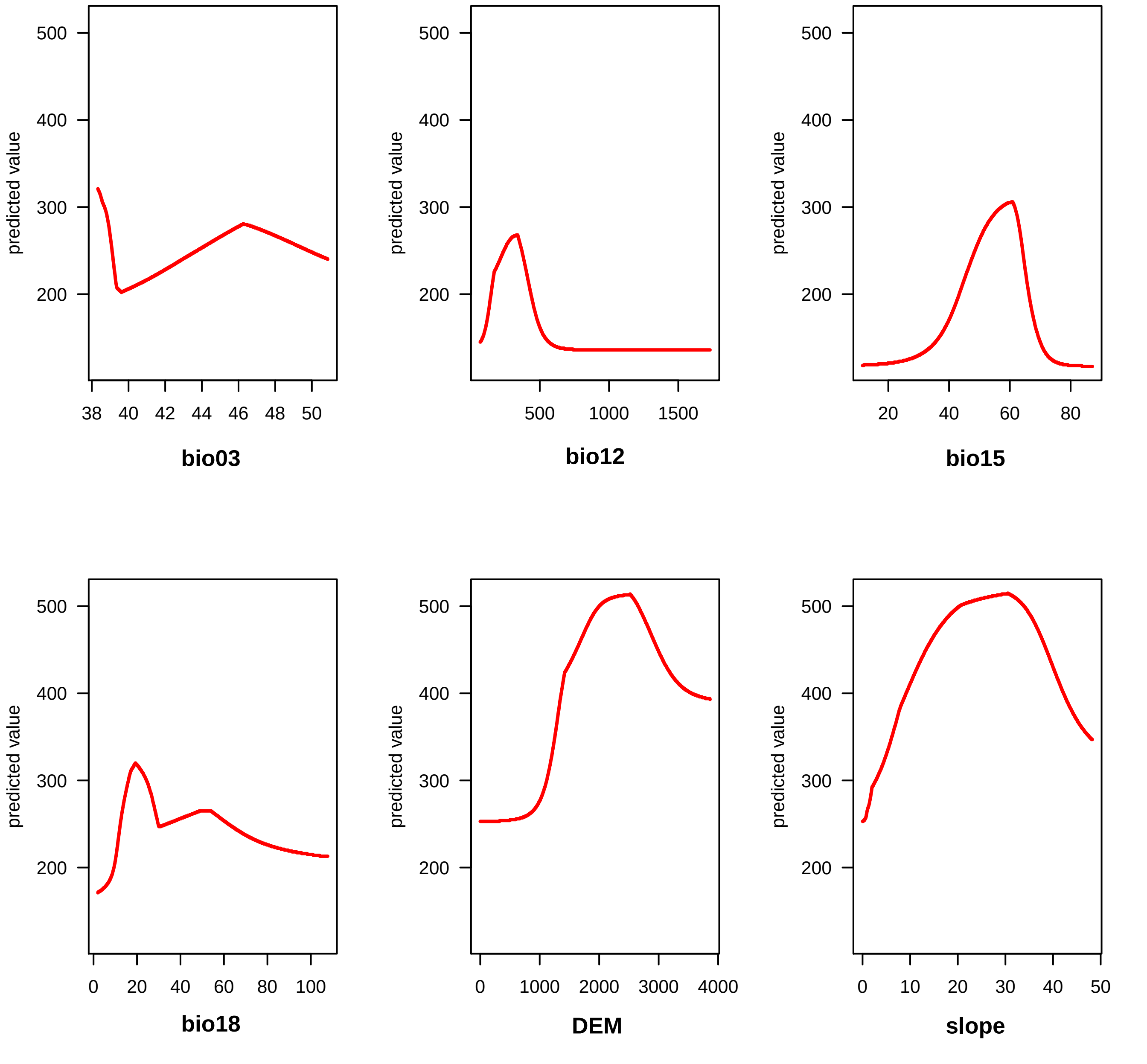

Figure S2.11. Response plots for ecoregion 14

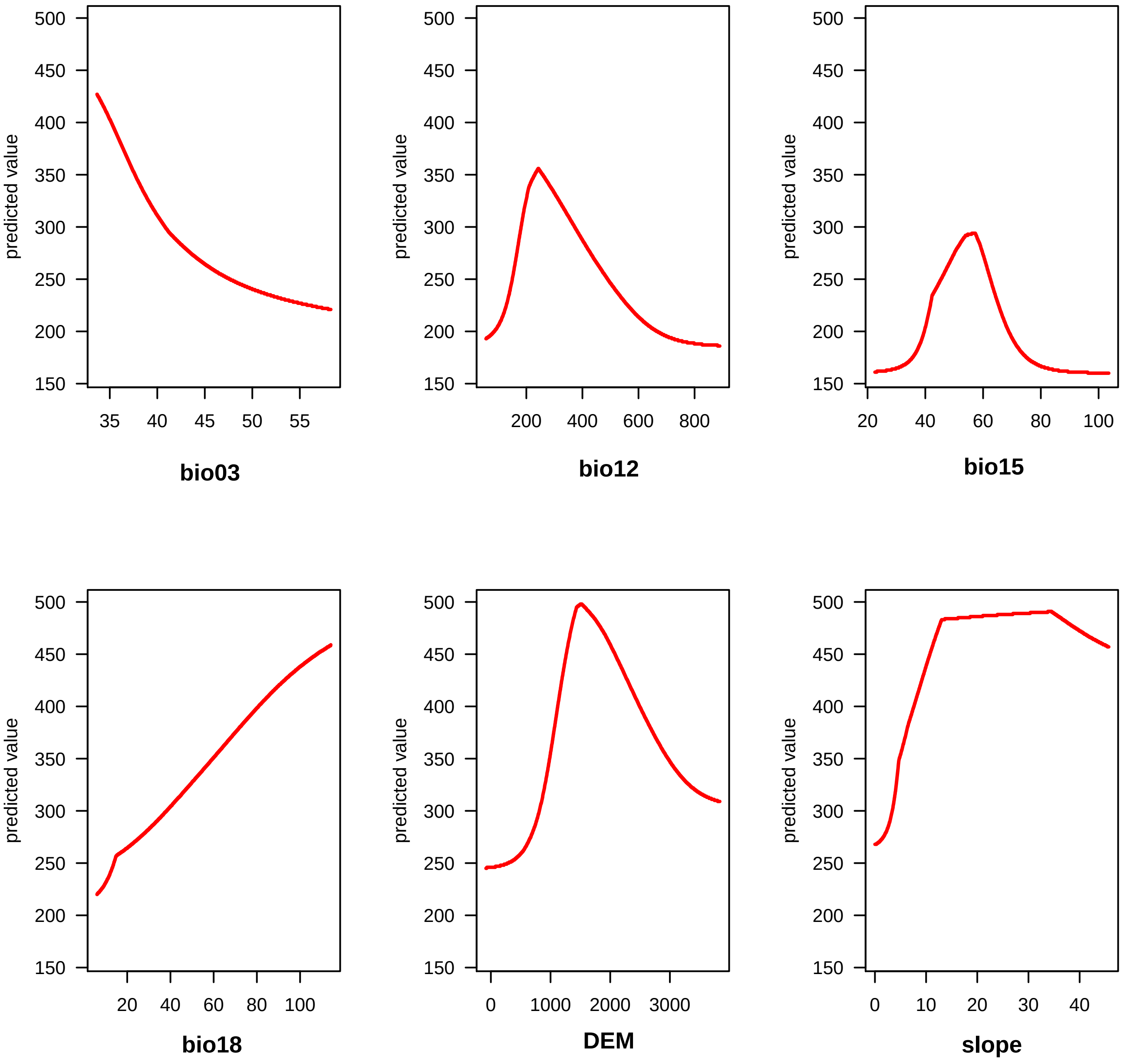

Figure S2.12. Response plots for ecoregion 78

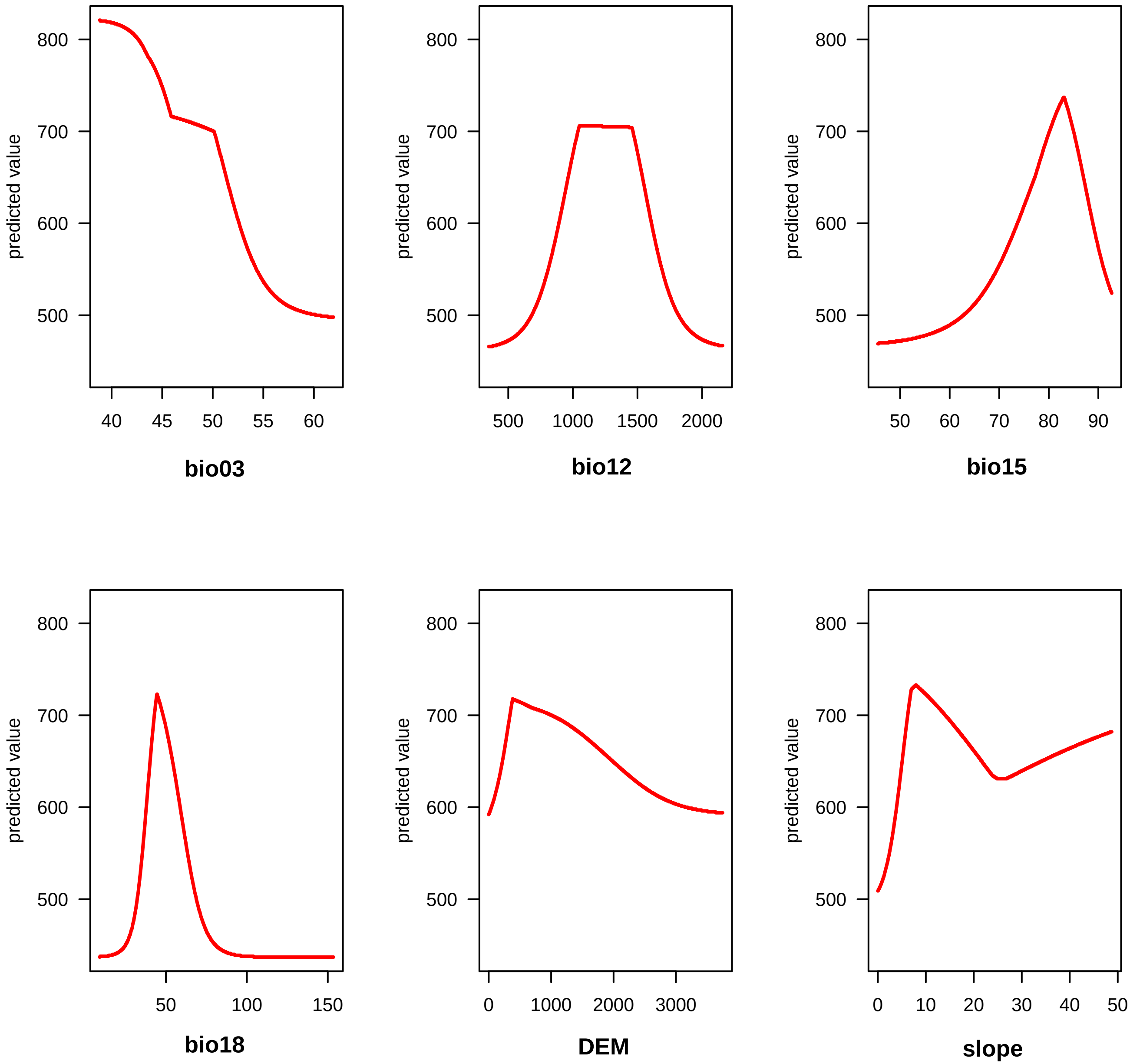

Figure S2.13. Response plots for ecoregion 85

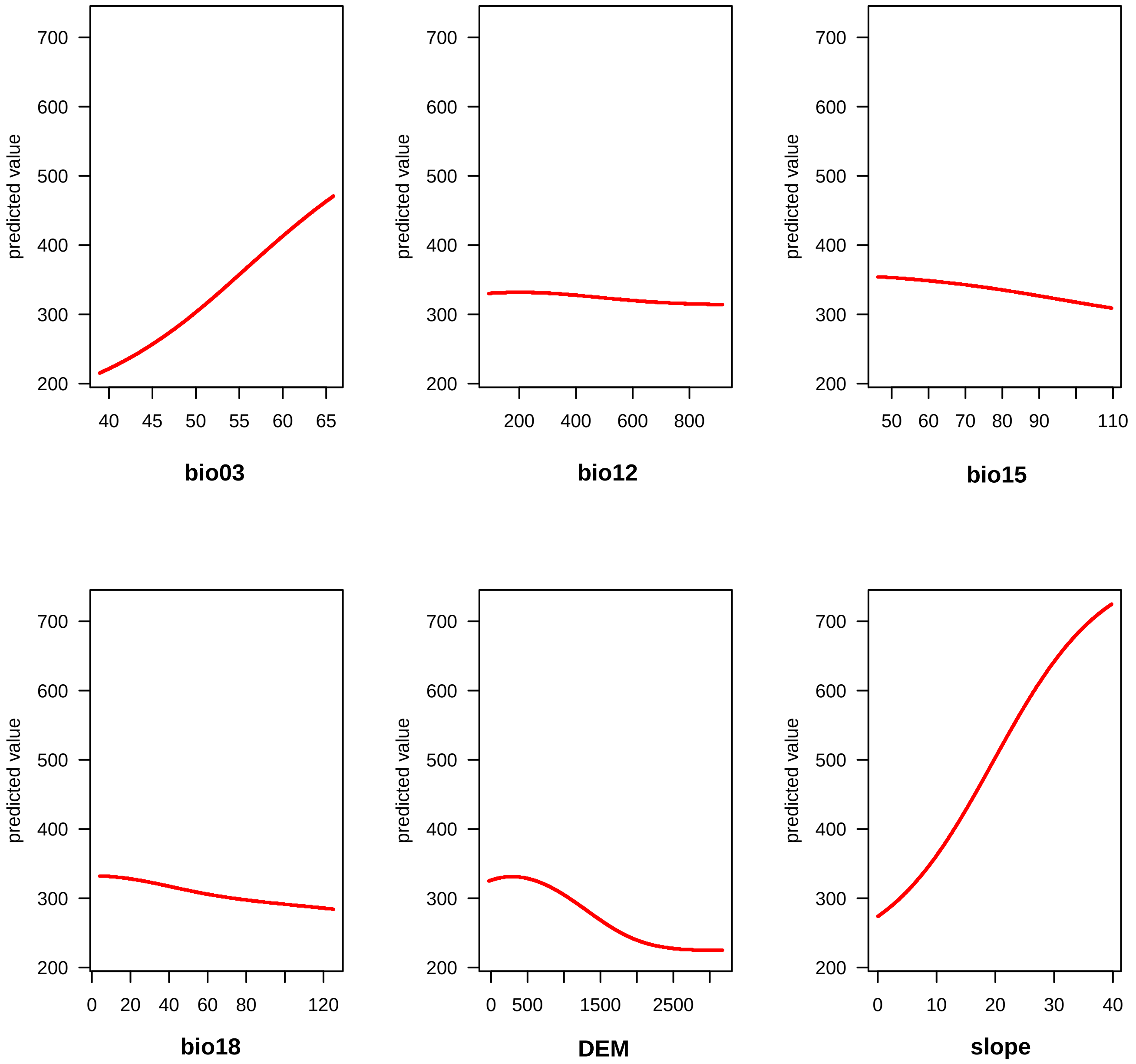

Supplement: Supplementary file 1 — Figures S1 [file ECE3-12-e9641-s002.pdf]
