# Supplementary material for: Predicting habitat suitability for Townsend's big‐eared bats across California in relation to climate change
Source: Ecol Evol. 2022 Dec 15;12(12):e9641. doi: 10.1002/ece3.9641 (PMC9755818; doi:10.1002/ece3.9641)
Supplement: Supplementary file 2 — Table S1 [file ECE3-12-e9641-s001.pdf]

Table S1.1

Data Level	Number of records	Description
Combined	241	All Occurrence Records
Transition	94	Non-breeding, non-hibernating roosts
Hibernacula	81	Winter roosts
Maternity	67	Summer roosts with >5 females roosting
Ecoregion 1	12	Coast Range
Ecoregion 4	21	Cascades
Ecoregion 5	23	Sierra Nevada
Ecoregion 6	37	Central California Foothills and Coastal Mountains
Ecoregion 7	0	Central California Valley
Ecoregion 8	20	Southern California Mountains
Ecoregion 9	8	Eastern Cascade Slopes and Foothills
Ecoregion 13	37	Central Basin and Range
Ecoregion 14	39	Mojave Basin and Range
Ecoregion 78	20	Klamath Mountains
Ecoregion 81	7	Sonoran Basin and Range
Ecoregion 85	11	Southern California/Northern Baja Coast

Table S1.1: Description of roost data included in this study. Data Level corresponds to state-wide and ecoregion-specific data, with the number of roosts found in each category. Description indicates what type of state-wide roosts are included, and the name of the level III ecoregions in California
