# Supplementary material for: Predicting habitat suitability for Townsend's big‐eared bats across California in relation to climate change
Source: Ecol Evol. 2022 Dec 15;12(12):e9641. doi: 10.1002/ece3.9641 (PMC9755818; doi:10.1002/ece3.9641)
Supplement: Supplementary file 3 — Table S2 [file ECE3-12-e9641-s004.pdf]

Table S1.2

Geographic Extent	AUC	TSS	Threshold	Null AUC	95% CI	p-value
All Occurrences	0.81	0.51	0.44	-	-	-
Intermediate	0.8	0.47	0.45	-	-	-
Hibernacula	0.86	0.57	0.43	-	-	-
Maternity	0.78	0.43	0.4	-	-	-
Ecoregion 1	0.92	0.88	0.54	0.67	0.90	1.31E-08
Ecoregion 4	0.98	0.91	0.38	0.77	0.97	2.97E-11
Ecoregion 5	0.90	0.80	0.63	0.77	0.89	2.01E-07
Ecoregion 6	0.85	0.64	0.51	0.74	0.84	1.10E-05
Ecoregion 8	0.98	0.95	0.39	0.66	0.97	9.30E-15
Ecoregion 13	0.98	0.93	0.45	0.70	0.97	2.66E-15
Ecoregion 14	0.9	0.73	0.37	0.72	0.88	1.25E-06
Ecoregion 78	0.8	0.6	0.57	0.75	0.85	4.28E-06
Ecoregion 85	0.89	0.83	0.39	0.72	0.78	0.002082

Table S1.2: Model performance for each of the models run in our study. AUC = Area under the receiver curve. AUC represents the mean AUC from 5-fold validation for state-wide models and 10-fold for ecoregion-specific models. MaxTSS = maximum True Skill Statistic, Threshold = maximized sum of sensitivity and specificity of the model. This is the threshold set for the creation of binary map for each model. Null AUC = The mean AUC value from 100 null models run with null model testing for our ecoregion-specific models. 95% CI = 95% One-sided 95% confidence interval for t.test comparing null model AUC to model AUC. p-value = p-value from our one-sided t-test, testing the alternative hypothesis true mean is greater than null.
