# Supplementary material for: Predicting habitat suitability for Townsend's big‐eared bats across California in relation to climate change
Source: Ecol Evol. 2022 Dec 15;12(12):e9641. doi: 10.1002/ece3.9641 (PMC9755818; doi:10.1002/ece3.9641)
Supplement: Supplementary file 4 — Table S3 [file ECE3-12-e9641-s003.pdf]

Table S1.3

	Bio03	Bio12	Bio15	Bio18	DEM	Slope
Combined	2.5424	31.33126	3.92612	**36.1294**	7.1108	18.96
Transition	2.9	**32.41404**	2.3266	**31.23004**	18.63114	13.68822
Maternity	5.44544	26.5663	5.24438	**37.00218**	17.89218	7.8495
Hibernacula	6.3935	21.87648	3.93754	14.21812	**33.16678**	20.40758
Ecoregion 1	0.9477221	1.02	0.95	**1.0829572**	0.99	1.01
Ecoregion 4	**1.0209974**	0.9800609	0.9911634	**1.0046279**	0.9990951	**1.0043745**
Ecoregion 5	1.0294416	0.9592813	1.0163638	**1.0740889**	0.9367018	0.9882539
Ecoregion 6	0.9836468	**1.0191214**	1.0132197	0.9971085	0.9681059	**1.0195384**
Ecoregion 8	**1.0084914**	0.9977668	0.9953172	0.9971383	0.9996223	1.0017008
Ecoregion 13	1.0104757	0.9038749	1.0089537	1.0182785	**1.0493431**	1.0132868
Ecoregion 14	1.0384014	0.8988841	1.0306774	**1.0594201**	0.9636364	1.0149783
Ecoregion 78	1.02604	1.002102	**1.039116**	0.89781	1.031864	1.007756
Ecoregion 85	0.8834957	1.1782754	1.0110559	0.8917618	**1.1866096**	0.8832556

Table S1.3. Contribution of each environmental predictor to the final model. For statewide models (Combined, Hibernacula, Maternity, and Transition), values indicate percent contribution to the model. For ecoregion-specific ensemble of small models output, values indicate proportional contribution of the variable in the final ensemble model. Value >1 indicates the focal variable has a higher contribution than average. Bold values indicate highest contributing variables.
